# The role of coupling among brain regions between the default mode network and the “Mesocircuit” Model in arousal in disorders of consciousness

**DOI:** 10.3389/fneur.2026.1829610

**Published:** 2026-08-04

**Authors:** Yi Li, Kangkang Bai, Jiayi Miao, Xuqiu Qin, Jiangli Cui, Qiao Shen, Yimin Zhou, Xingyu Miao

**Affiliations:** 1 Department of Neurosurgery, Shaanxi Provincial People's Hospital, Xi'an, Shaanxi, China; 2 Xi'an Medical University, Xi'an, Shaanxi, China

**Keywords:** “Mesocircuit” Model, default mode network, disorders of consciousness, external globus pallidus, medial prefrontal cortex, posterior cingulate cortex

## Abstract

**Introduction:**

This study examined the association between inter-regional functional connectivity (FC) strength and arousal, and explored how interactions between the default mode network (DMN) and the brain regions of the “Mesocircuit” Model influence consciousness generation and recovery in patients with disorders of consciousness (DoC).

**Methods:**

Thirty DoC patients [eight in coma, 10 in vegetative state (VS), 12 in minimally conscious state (MCS)] from the Department of Neurosurgery, Shaanxi Provincial People's Hospital underwent resting-state functional magnetic resonance imaging. Seed regions included DMN cores [posterior cingulate cortex (PCC), medial prefrontal cortex (mPFC) and the “Mesocircuit” Model core external globus pallidus (GPe)]; FC and correlation analyses were performed.

**Results:**

Results showed that compared with VS/MCS groups, the coma group had weaker FC between PCC and left anterior cingulate gyrus/right middle temporal gyrus/GPe, and between mPFC and right superior frontal gyrus/left supramarginal gyrus/GPe, but stronger FC between mPFC/PCC and bilateral central lateral thalamus (CL). For GPe-seeded FC, the coma group had reduced FC with DMN nodes (PCC/mPFC) and left CL, but increased FC with right CL. Correlation analysis revealed positive associations of PCC-mPFC, PCC-GPe, mPFC-GPe, and GPe-CL_L FC with CRS-R/GCS scores, and negative associations of PCC-CL, mPFC-CL, and GPe-CL_R FC with these scores.

**Conclusion:**

The functional integrity of the DMN and the “Mesocircuit” Model, as well as the FC between their brain regions, are closely related to the consciousness level of patients with DoC. PCC-GPe, PCC-mPFC, GPe-CL_L, and PCC-CL FC may serve as neurobiological markers for DoC diagnosis and prognosis, aiding precise management.

## 1 Introduction

DoC refer to abnormal changes in wakefulness, content of consciousness, and scope of awareness. These disorders result from damage or functional suppression of key brain regions. These regions include the cerebral cortex, subcortical structures, and the brainstem reticular formation (1). From a clinical perspective, disorders of consciousness can be divided into different types. Coma is the most severe form. In this state, patients experience a complete loss of consciousness. They do not respond to external stimuli and show no voluntary movement. Their brainstem reflexes may be present or absent (2–4). Patients in a vegetative state (VS) can open their eyes and have sleep-wake cycles. They show no signs of conscious awareness and lack awareness of themselves and their surroundings (5, 6). Patients in a minimally conscious state (MCS) show irregular and weak, but clear, signs of conscious behaviors. Examples include following commands or responding to words (7, 8). The pathophysiological mechanisms of DoC involve abnormalities in multiple neurobiological processes. It is widely believed that structural and functional damage of the brain is an important basis for DoC (9). Severe brain injuries, such as trauma or hypoxia-ischemia, can cause neuronal death, axonal damage, and abnormal responses in glial cells. These changes can disrupt the integrity of neural circuits (10–13). A study by Lutkenhoff et al. (14) shows that atrophy in “Mesocircuit” Model areas such as the thalamus and basal ganglia is often observed in patients with DoC. These areas are closely linked to the maintenance and regulation of consciousness. A study by Bodart et al. (15) shows a significant correlation between global structural integrity and FC in patients with DoC. More structural damage reduces FC, which may impair consciousness. A study by Bodien et al. (16) also shows that patients with DoC have functional network connectivity during rest that is different from healthy individuals. This is especially true in the DMN and other cortical and subcortical networks. These changes in functional connectivity may be closely related to the severity of DoC and recovery (17).

The “Mesocircuit” Model and the DMN play a key role in DoC. Patients with DoC often show functional and structural abnormalities in these networks (18, 19). A study by Crone et al. (20) supports the “Mesocircuit” Model hypothesis. This idea suggests that neural differentiation between the frontal cortex, subcortical areas, and the thalamus is key to explaining the recovery of consciousness. Our previous study showed that abnormal FC between the GPe and cortical regions is also closely related to the arousal state of patients. This further supports the role of “Mesocircuit” Model hypothesis in the regulation of consciousness (21). A study by Seo et al. (22) used fMRI imaging in patients with DoC. They found that FC between the thalamus and the DMN is significantly reduced. This was especially notable in the mPFC, PCC and precuneus regions. This damage impairs the self-inhibitory function of these areas. These connections may be involved in information transfer and functional coordination between the “Mesocircuit” Model and the DMN. The coupling between the “Mesocircuit” Model and the DMN in the brain is crucial for maintaining normal conscious states (23, 24). In patients with DoC, these regions and their connections may be damaged, which can affect the “Mesocircuit” Model-DMN coupling, leading to disorders of consciousness (19).

This study uses rs-fMRI to examine patients with different subtypes of DoC. Using pre-defined seed points, it aims to explore the relationship between the coupling of the DMN and the “Mesocircuit” Model and different conscious states, and further to analyze the association between FC strength among brain regions and arousal. This study aims to reveal the mechanisms by which multiple network interactions influence the generation and recovery of consciousness in the context of DoC.

## 2 Materials and methods

### 2.1 Participants

This retrospective study was conducted utilizing clinical data from 30 patients with DoC. These patients were admitted to the Department of Neurosurgery at Shaanxi Provincial People's Hospital between November 2023 and May 2025.Patients were excluded from the study based on the following criteria: (1) presence of any contraindications for MRI scanning, such as a body temperature higher than 39.0 °C within the past 3 days or implanted metallic devices; (2) a documented history of psychiatric or neurological disorders; or (3) administration of sedatives or neuromuscular blocking agents prior to the examination. The collected clinical data encompassed the following categories: (1) demographic information, including sex, age, etiology, lesion characteristics, and time since onset; (2) behavioral assessment scales, specifically the Glasgow Coma Scale (GCS) and the Coma Recovery Scale-Revised (CRS-R); and (3) neuroimaging data, including structural images (3D T1-weighted) and functional images (resting-state blood-oxygen-level-dependent functional MRI, BOLD-fMRI). Participants were categorized into three groups based on their diagnosis: coma group (*n* = 8), VS group (*n* = 10), and MCS group (*n* = 12). The detailed clinical characteristics of the enrolled patients are summarized in Table 1.

**Table 1:** Detailed table demographic and clinical characteristics of DoC patients.

Patient ID	Sex	Age	Etiology	Course	CRS-R on day of scan	Max CRS-R on week of scan	GCS on day of scan	Max GCS on week of scan	Diagnosis
1	F	49	Non-traumatic brain injury	123	6	6	8	8	MCS
2	F	56	Traumatic brain injury	5	1	1	3	3	Coma
3	M	61	Traumatic brain injury	7	1	1	3	3	Coma
4	F	60	Non-traumatic brain injury	10	1	1	3	3	Coma
5	M	55	Traumatic brain injury	33	10	10	7	7	MCS
6	F	65	Non-traumatic brain injury	30	4	4	6	6	VS
7	M	73	Non-traumatic brain injury	35	6	6	7	7	MCS
8	M	79	Non-traumatic brain injury	40	7	7	8	8	MCS
9	M	59	Non-traumatic brain injury	40	5	5	6	6	VS
10	M	50	Traumatic brain injury	5	0	0	3	3	Coma
11	F	25	Traumatic brain injury	68	8	9	8	8	MCS
12	M	40	Traumatic brain injury	38	7	7	8	8	MCS
13	M	36	Non-traumatic brain injury	10	7	6	5	5	VS
14	M	35	Non-traumatic brain injury	28	5	5	6	6	VS
15	F	74	Non-traumatic brain injury	7	1	1	3	3	Coma
16	F	60	Non-traumatic brain injury	55	6	6	8	8	MCS
17	F	66	Non-traumatic brain injury	38	6	6	5	5	VS
18	F	75	Non-traumatic brain injury	29	6	6	6	6	VS
19	M	68	Non-traumatic brain injury	13	1	1	3	3	Coma
20	M	36	Traumatic brain injury	25	8	8	7	7	VS
21	F	29	Non-traumatic brain injury	33	11	11	10	10	MCS
22	M	43	Non-traumatic brain injury	44	4	4	6	6	VS
23	F	35	Non-traumatic brain injury	5	7	7	8	8	MCS
24	F	52	Non-traumatic brain injury	29	1	1	1	1	Coma
25	M	46	Non-traumatic brain injury	46	6	6	8	8	MCS
26	M	77	Traumatic brain injury	18	9	9	8	8	MCS
27	M	68	Non-traumatic brain injury	22	2	2	3	3	Coma
28	M	64	Non-traumatic brain injury	38	6	6	6	6	VS
29	F	39	Non-traumatic brain injury	21	5	5	7	7	VS
30	F	46	Traumatic brain injury	10	10	10	8	8	MCS

F, female; M, male; CRS-R, Coma Recovery Scale–Revised; GCS, Glasgow Coma Scale; Coma, coma patients; VS, vegetative state patients; MCS, minimally conscious state patient.

On the day of MRI scanning, each patient enrolled in this study underwent clinical evaluation and diagnosis by a trained clinical neurosurgeon using the CRS_R and the GCS. Ethical approval was obtained from the Ethics Committee of Shaanxi Provincial People's Hospital. Written informed consent was acquired from all legal guardians of the enrolled patients at least 24 h prior to the neuroimaging examinations.

### 2.2 Evaluation of level of consciousness and arousal

All participants underwent standardized clinical evaluations using the CRS–R (25) and the GCS (26) to determine their level of consciousness. The assessments were conducted by two experienced neurosurgeons according to the following protocol: evaluations were performed three times per day, every other day, beginning 1 week prior to the MRI scan, and also on the day of scanning. The highest score obtained across all evaluation time points was recorded for each patient. Full details are shown in Table 2.

**Table 2:** Diagnostic criteria for DoC based on behavioral assessment scale.

Scale	Coma	VS	MCS
CRS-R		Auditory ≤ 2 and	Auditory =3–4or
		Visual ≤ 1 and	Visual =2–5or
		Motor ≤ 2 and	Motor =3–5or
		Oromotor/verbal ≤ 2 and	Oromotor/verb = 3 or
		Communication = 0 and	Communication = 1
	Arousal=0	Arousal = 1–2	
GCS	Eye =1	Eye =2–4 and	Verbal =3–5 or
		Verbal =1–2 and	Motor =5–6
		Motor =1–4	

### 2.3 MRI data acquisition

All structural and functional MRI data were acquired using a 3.0T Philips Ingenia CX MRI scanner (Philips Healthcare, The Netherlands) with a 32-channel phased-array head coil at Shaanxi Provincial People's Hospital. During scanning, all participants were instructed to lie quietly in a supine position with their heads comfortably stabilized using foam padding to minimize motion artifacts. Earplugs were provided to reduce scanner noise and enhance patient comfort. High-resolution T1-weighted structural images were obtained using a three-dimensional fast spoiled gradient echo (3D-FSPGR) sequence with the following parameters: repetition time (TR) = 8.2 ms; echo time (TE) = 3.8 ms; field of view (FOV) = 220 × 220 mm^2^; matrix size = 224 × 224; slice thickness = 1 mm; slice gap = 0 mm; number of slices = 190.Resting-state functional MRI data were collected using a gradient-echo echo-planar imaging (GRE-EPI) sequence with the following parameters: TR = 2,000 ms; TE = 30 ms; FOV = 240 × 240 mm^2^; matrix = 80 × 80; flip angle = 90°; number of time points = 200; slice thickness = 4 mm; number of slices = 34.

### 2.4 Preprocessing of resting-state fMRI data

In this study, preprocessing of both structural 3D T1-weighted and resting-state functional MRI data was performed using the Data Processing & Analysis for Brain Imaging (DPABI, v5.4, http://rfmri.org/DPABI) toolbox, specifically the DPARSF module (27). The preprocessing pipeline implemented in DPARSF is built upon algorithms from Statistical Parametric Mapping 12 (SPM12, http://www.fil.ion.ucl.ac.uk/spm). The detailed steps were as follows: the first 10 volumes of each functional scan were discarded to allow for magnetic field stabilization. The remaining images underwent slice timing correction to account for inter-slice acquisition delays, followed by realignment to correct for head motion. Participants exhibiting excessive head motion (mean framewise displacement > 0.2 mm) were excluded from subsequent analyses. The functional images were then co-registered to the individual high-resolution T1-weighted structural image. After nuisance signal regression (including white matter, cerebrospinal fluid, and global mean signal), the images were normalized to the standard Montreal Neurological Institute (MNI) space using the T1 template and resampled to an isotropic resolution of 3 mm^3^. Finally, spatial smoothing was applied using a Gaussian kernel with a full width at half maximum (FWHM) of 4 mm.

### 2.5 Selection of regions of interest (ROI)

To functionally characterize brain regions for each participant, seed-based functional connectivity analysis was conducted. Two ROIs were selected as seeds for the DMN: the mPFC and PCC (19). Additionally, four ROIs were chosen to represent the Mesocircuit model: the bilateral external globus pallidus (GPe) and the bilateral central lateral thalamic nuclei (CL), the bilateral GPe was selected as the seed region for systematic functional connectivity evaluation. For each seed region, the mean time series was extracted and correlated with the time series of every voxel across the whole brain to generate individual functional connectivity.

Using HCPex template, this template can rely on access to the link (https://github.com/wayalan/HCPex/tree/main) to obtain, has developed an integrated mask, this integrated mask is specifically designed for bilateral GPe, PCC, and mPFC. This mask is used as a seed region to conduct the assessment of functional connectivity. It is necessary to calculate what the functional connectivity index is between each seed region and all voxels in the entire brain. The Fisher Z-transform needs to be performed on the relevant values to convert them into *Z*-scores. This transformation is carried out to enhance the normality of the data. The *Z*-score we obtained can indicate the strength of the functional connection between each seed area and the rest of the brain.

### 2.6 Statistical analysis

Statistical analyses were performed using SPSS (version 25.0; IBM Corp, Armonk, NY, USA). Data are presented as frequencies (counts), medians, percentages, or interquartile ranges. Categorical variables were compared between groups using the chi-square test. Continuous data are expressed as mean ± standard deviation. Prior to group comparisons, the normality of the data distribution was assessed using the Shapiro–Wilk test, and homogeneity of variances was verified with Levene's test. For data satisfying both normality and homogeneity of variance, one-way analysis of variance (ANOVA) was employed to evaluate between-group differences. If data were normally distributed but variances were unequal, Welch's corrected *t*-test was applied. For data that did not follow a normal distribution, non-parametric tests were used. A *P*-value of less than 0.05 was considered statistically significant.

Resting-state fMRI data were subjected to statistical analysis utilizing DPABI software and the CONN toolbox (28, 29). The analysis focused on evaluating differences in functional connectivity (FC) strength across the coma, VS, and MCS groups. To address the issue of multiple comparisons, the Gaussian Random Field (GRF) method was employed, applying a dual-threshold criterion with voxel-level significance set at *P* < 0.001 and cluster-level significance at *P* < 0.05. Correlation matrices were constructed utilizing the Spearman correlation method. To assess relationships involving the dichotomous arousal state variable, point-biserial correlation analysis was specifically applied (30, 31). Statistical significance levels, adjusted using the Benjamini–Hochberg false discovery rate (FDR) correction, are indicated by asterisks as follows: ^***^ for *P* < 0.001, ^**^ for *P* < 0.01, and ^*^ for *P* < 0.05, Non-significant correlations *P* ≥ 0.05 are shown without asterisks.

## 3 Results

### 3.1 Demographic and behavioral scale results

This study enrolled 30 patients with disorders of consciousness [16 males, 14 females; nine traumatic brain injury (TBI), 21 non-traumatic brain injury (NTBI)]. Patients were stratified into three subgroups based on standardized assessments using the CRS-R and GCS. Coma group (*n* = 8; four males, four females; three TBI, five NTBI); VS group (*n* = 10; six males, four females; one TBI, nine NTBI) and MCS group (*n* = 12; six males, six females; five TBI, seven NTBI). Statistical analyses revealed no significant differences in age (*P* = 0.904), sex (*P* = 0.290), Mean FD (*P* = 0.857) or etiology (*P* = 0.248) distribution among the three patient groups (coma, VS, and MCS). In contrast, significant correlations were identified between the time since onset (*P* = 0.005) and scores on the GCS (*P* = 0.000) as well as the CRS–R (*P* = 0.000). Detailed results are presented in Table 3.

**Table 3:** Demographic and clinical characteristics of DoC patients.

Variables	Coma (*n* = 8)	VS (*n* = 10)	MCS (*n* = 12)	*P*-value
Sex (female/male)	4/4	6/4	6/6	0.904^a^
Age (mean ± SD)	61.125 ± 8.408	51.8 ± 15.411	51.17 ± 18.17	0.290^b^
Etiology *n* (%)				0.248^a^
TBI	3 (37.5%)	1 (10%)	5 (41.7%)	
NTBI	5 (62.5%)	9 (90%)	7 (58.3%)	
Course M (Q1, Q3)	8.5 (5.5, 19.75)	29.5 (24.00, 38.50)	36.5 (21.75, 52.75)	0.005^b^
CRS-R M (Q1, Q3)				
Day of scan	1 (1.00, 1.00)	5.6 (4.75.6.25)	7.75 (6.00, 9.75)	0.000^b^
Max	1 (1.00, 1.00)	5.5 (4.75.6.00)	7.83 (6.00, 9.75)	0.000^b^
GCS M (Q1, Q3)				
Day of scan	2.75 (3.00, 3.00)	6 (5.75, 6.25)	8 (8.00, 8.00)	0.000^b^
Max	2.75 (3.00, 3.00)	6 (5.75, 6.25)	8 (8.00, 8.00)	0.000^b^
Mean FD (mm)	0.092 ± 0.041	0.088 ± 0.037	0.085 ± 0.039	0.857

TBI, traumatic brain injury; NTBI, non-traumatic brain injury; course, symptom onset to scan interval; max, max on week of scan; mean FD, mean framewise displacement. No significant intergroup difference in head motion was observed among the three groups. Statistical values are expressed as mean ± standard deviation.

^a^*P*-value is obtained from chi–square test analysis.

^b^*P*-value is obtained from K-W test.

### 3.2 Results of the functional connectivity analysis

#### 3.2.1 Resting-state ROI-to-ROI functional connectivity analysis

**Figure 1:**
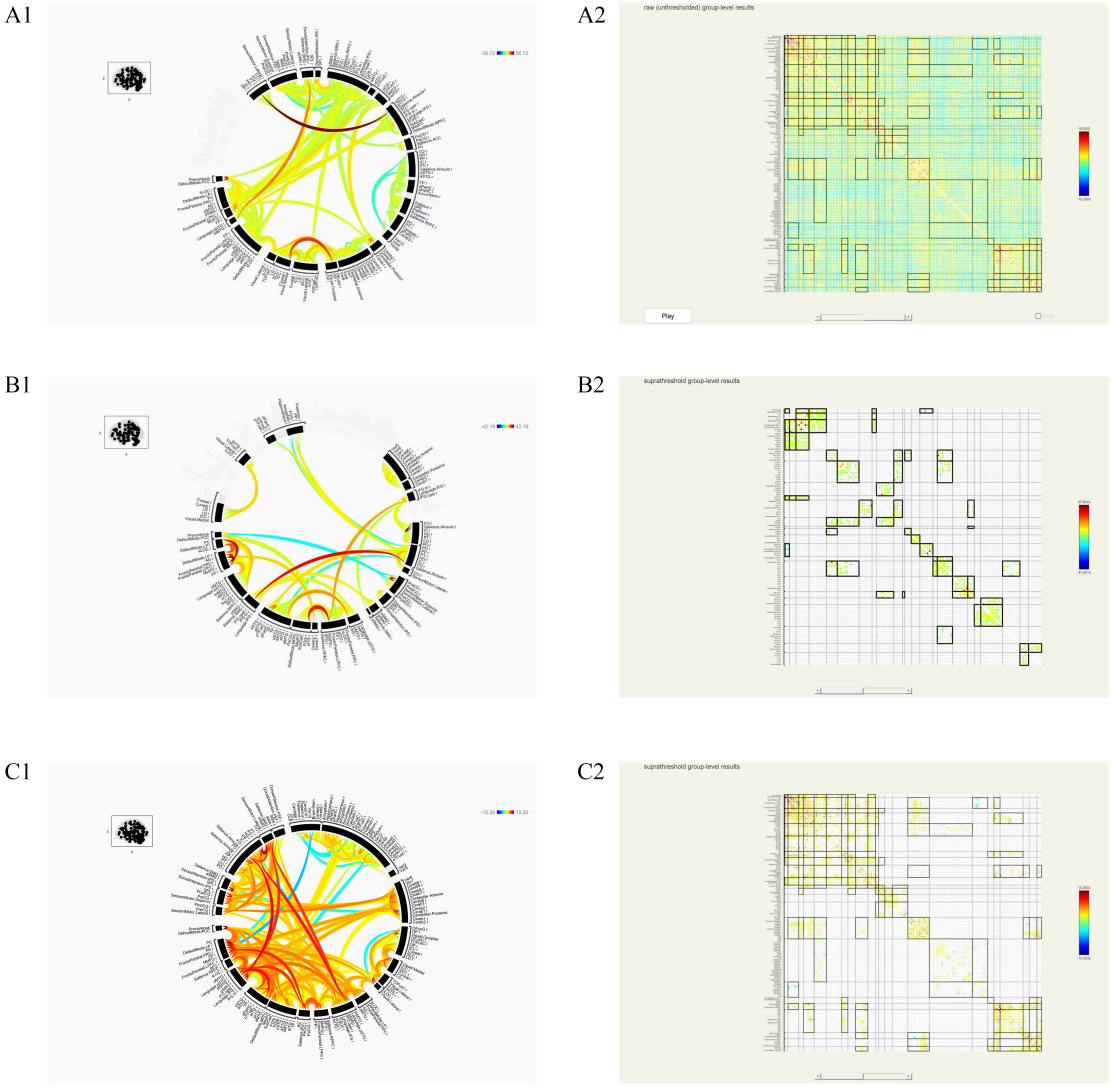
Resting-state ROI-to-ROI functional connectivity analysis and connectivity matrix representation among patient groups in coma/VS/MCS. Comparative alterations in interregional functional connectivity strength and connectivity matrices between coma, VS and MCS patient groups. **(A1, A2)**: Coma vs. VS; **(B1, B2)**: Coma vs. MCS; **(C1, C2)**: VS vs. MCS. Red indicates increased functional connectivity strength, while blue indicates decreased functional connectivity strength. On the right side, black squares stand for brain regions; blue and red refer to weak and strong functional connectivity, respectively. In the left panel, red means elevated functional connectivity strength, blue means reduced strength, and the transitional yellow color indicates no significant intergroup difference.

Results from whole-brain ROI-to-ROI functional connectivity analyses, conducted through pairwise comparisons between the three patient groups (Coma, VS, and MCS). Compared to the coma group, both the VS group and the MCS group showed a general trend of increased FC. The MCS group showed the strongest increase. The most significant changes in functional connectivity were observed between the PCC and the mPFC (Figure 1).

#### 3.2.2 Analysis of absolute ALFF

**Figure 2:**
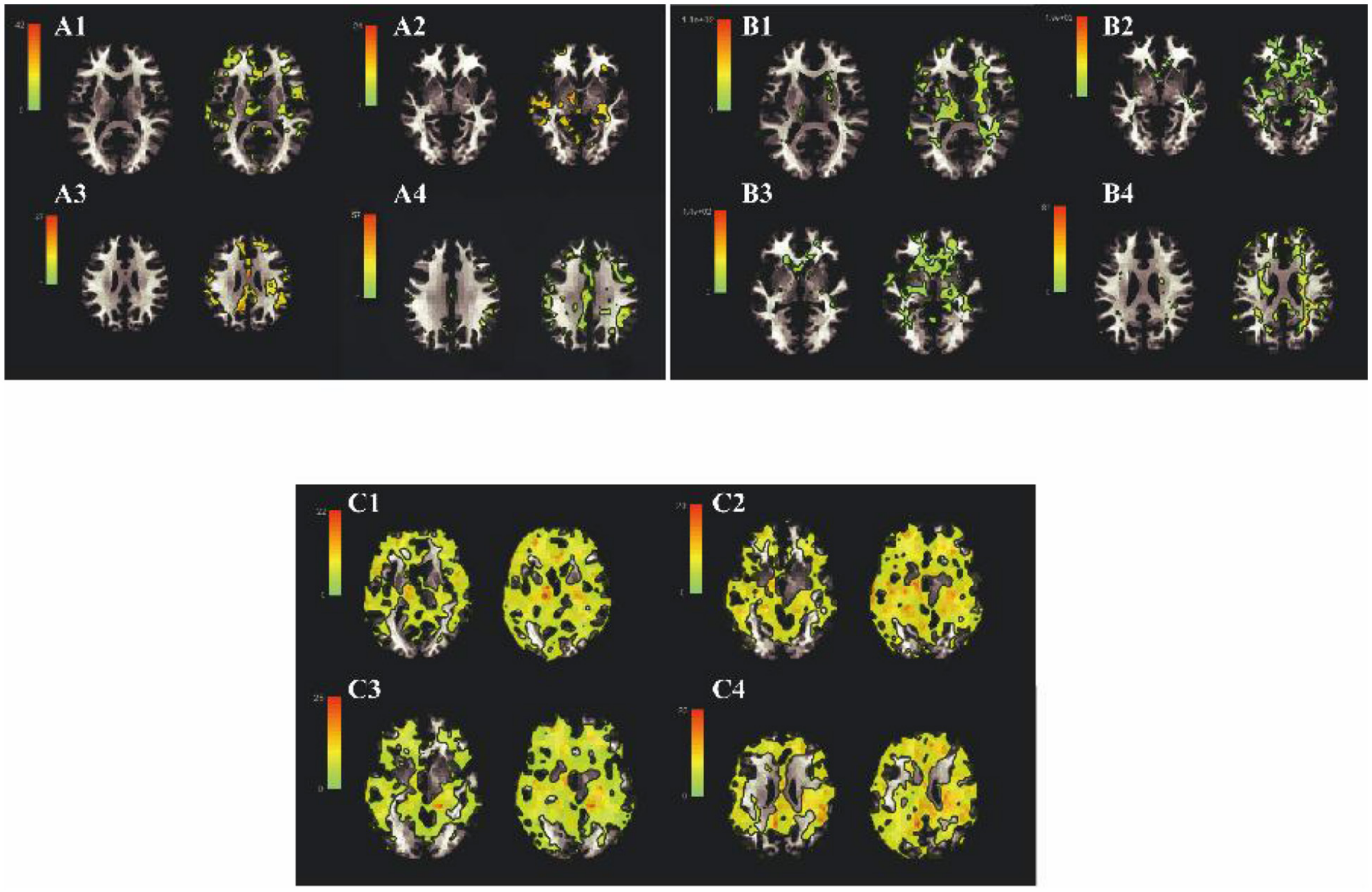
Absolute ALFF variations in the CL, GPe, mPFC, and PCC among coma/VS/MCS patient groups. **(A)** ALFF alterations in coma group vs. VS group. **(A1)** CL; **(A2)** GPe; **(A3)** mPFC; **(A4)** PCC. **(B)** ALFF alterations in coma group vs. MCS group. **(B1)** CL; **(B2)** GPe; **(B3)** mPFC; **(B4)** PCC. **(C)** ALFF alterations in VS group vs. MCS group. **(C1)** CL; **(C2)** GPe; **(C3)** mPFC; **(C4)** PCC. Warm colors (red) reflect increased ALFF; cool colors (green) represent decreased ALFF.

ALFF quantifies the intensity or amplitude of low-frequency fluctuations (typically 0.01–0.1 Hz) in the BOLD signal during rest. It is considered a reliable metric for assessing the magnitude of spontaneous neural activity within a specific brain region. Increased ALFF was typically interpreted as reflecting local neural hyperactivation or elevated neuronal excitability, while decreased ALFF indicated the opposite pattern. We found significant alterations in the absolute ALFF within the predefined regions of interest. Compared with patients in the coma group, individuals in both the VS and MCS groups exhibited significantly increased absolute ALFF values in the bilateral CL, GPe, mPFC, and PCC. In contrast, the VS group demonstrated significantly lower absolute ALFF in the pre-defined regions of interest relative to the MCS group (Figure 2).

The absolute ALFF values of bilateral CL, GPe, mPFC and PCC exhibited significant group differences among the three cohorts (all *P* < 0.001), with large effect sizes [partial η^2^ = 0.62–0.67, 95% CI (0.37, 0.79)]. *Post-hoc* pairwise comparisons revealed that the coma group had significantly lower ALFF than the VS and MCS groups, and the VS group showed reduced ALFF relative to the MCS group [*r* = 0.58–0.78, 95% CI (0.27, 0.91), all *P* < 0.001; see Table 4 and 5].

**Table 4:** Overall one-way analysis of variance for ALFF across three groups.

ROI	*F*	Partial η^2^	95% CI	*P*-value
CL	*F* = 28.62	0.67	[0.42, 0.79]	< 0.001
GPe	*F* = 26.15	0.64	[0.39, 0.77]	< 0.001
mPFC	*F* = 24.89	0.62	[0.37, 0.76]	< 0.001
PCC	*F* = 27.01	0.65	[0.40, 0.78]	< 0.001

**Table 5:** ALFF pairwise comparisons.

Intergroup comparison	Brain region	*r*	95% CI	*P*-value
Coma vs. VS	CL	0.71	[0.43, 0.87]	< 0.001
Coma vs. VS	GPe	0.68	[0.39, 0.85]	< 0.001
Coma vs. VS	mPFC	0.66	[0.36, 0.84]	< 0.001
Coma vs. VS	PCC	0.69	[0.40, 0.86]	< 0.001
Coma vs. MCS	CL	0.78	[0.54, 0.91]	< 0.001
Coma vs. MCS	GPe	0.75	[0.49, 0.89]	< 0.001
Coma vs. MCS	mPFC	0.73	[0.46, 0.88]	< 0.001
Coma vs. MCS	PCC	0.76	[0.50, 0.90]	< 0.001
VS vs. MCS	CL	0.62	[0.31, 0.82]	< 0.001
VS vs. MCS	GPe	0.6	[0.29, 0.81]	< 0.001
VS vs. MCS	mPFC	0.58	[0.27, 0.79]	< 0.001
VS vs. MCS	PCC	0.61	[0.30, 0.82]	< 0.001

#### 3.2.3 FC analysis using the PCC as a seed region

**Figure 3:**
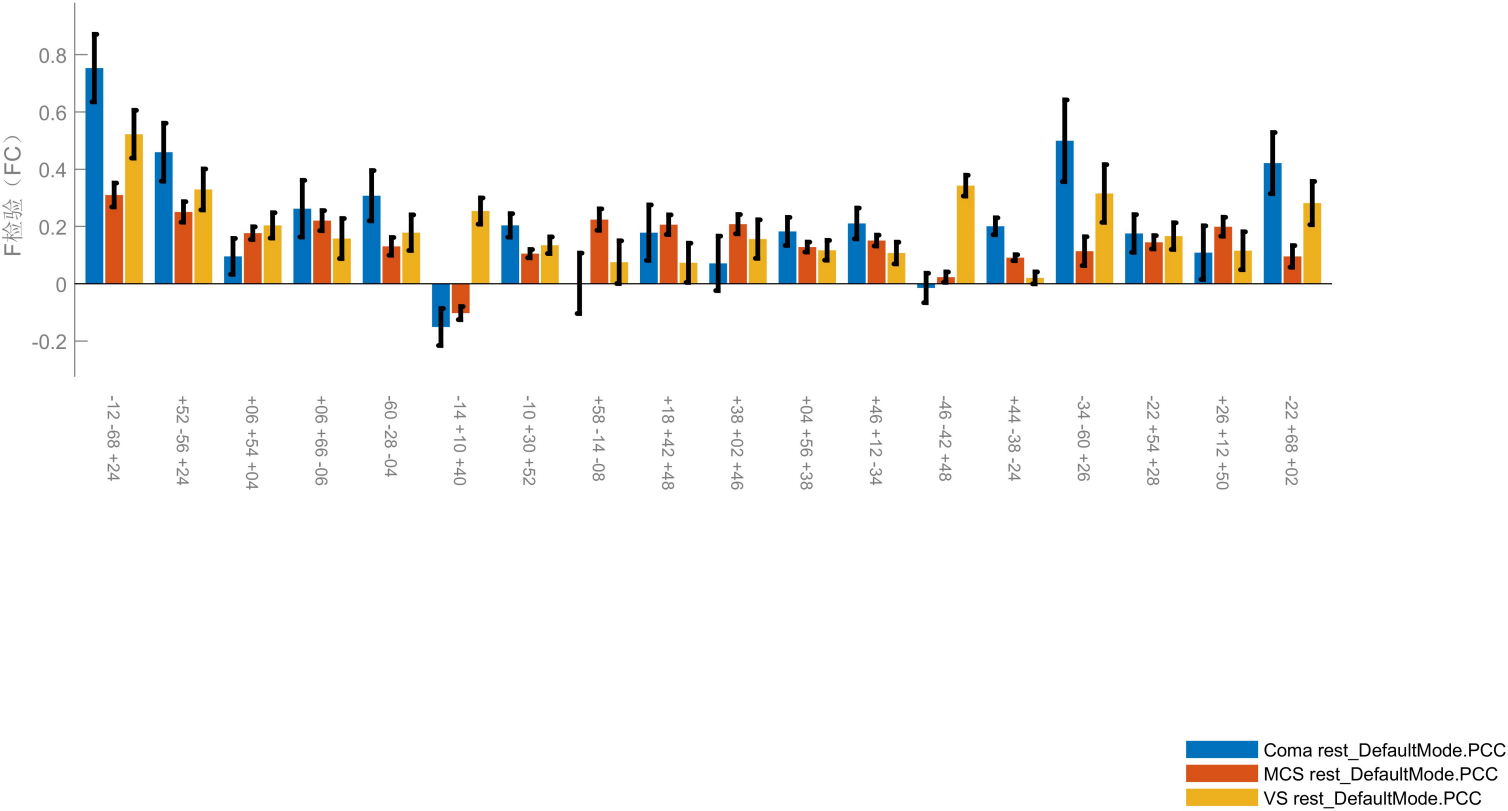
PCC-seeded FC differences a coma/VS/MCS patient groups in significantly altered brain regions. (−12 −68 +24): Lingual L; (+52 −56 +24): Temporal sup R; (+6 +54 +04): Frontal sup Medical R; (+06 −66 +06): Lingual R; (+60 −28 −04): Temporal Mid R; (−14 +10 +40): Cingulum ant L; (−10 +32 +52): Frontal sup Medical L; (+58 −14 −08): Temporal Mid R; (+18 +42 +48): Frontal sup R; (+38 +02 +46): Precentral R; (+4 +56 +38): Frontal sup Medical R; (+46 +12 −34): Temporal pole Mid R; (−46 −42 +48): Parietal inf L; (+44 −38 −24): Fusiform R; (−34 −60 +26): Occipital Mid L; (−22 +54 +28): Frontal sup L: (+26 +12 +50): Frontal Mid R. More pronounced *F*-values indicate greater differences in functional network organization across clinical groups (e.g., coma, VS, MCS).

**Figure 4:**
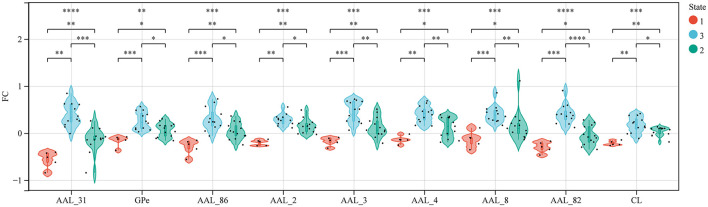
FC between the PCC and specific brain regions across coma, VS, and MCS groups. Violin plots depict the distribution of FC between PCC and nine specific brain regions across groups. AAL_31: Cingulum Ant_L; Gpe: Globus Pallidus externa; AAL_86: Temporal_Mid_R; AAL2:Precentral_R; AAL3: Frontal_Sup_L; AAL4: Frontal_Sup_R; AAL8: Frontal_Mid_R; AAL82: Temporal_Sup_R; CL: Thalamus Central lateral. Differences in FC among the three groups were analyzed using non-parametric tests. ****P < 0.0001; ***P < 0.001; **P < 0.01; *P < 0.05. State: (1) Coma; (2) VS; (3) MCS.

Comparative analyses using the PCC as the seed region across three groups revealed that comatose patients exhibited significantly reduced functional connectivity strength compared to both the vegetative state and minimally conscious state groups. Specifically, decreased functional connectivity was observed between the PCC and the following regions: the left anterior cingulate gyrus (AAL_31: Cingulum_Ant_L), the right middle temporal gyrus (AAL_86: Temporal_Mid_R), the right precentral gyrus (AAL2: Precentral_R), the left superior frontal gyrus (AAL3: Frontal_Sup_L), the right superior frontal gyrus (AAL4: Frontal_Sup_R), the right middle frontal gyrus (AAL8: Frontal_Mid_R), and the right superior temporal gyrus (AAL82: Temporal_Sup_R). These results survived Gaussian random field correction at a voxel-level threshold of *P* < 0.001 and a cluster-level threshold of *P* < 0.05. To explore the relationship between the coupling of the DMN and the “Mesocircuit” model with disorders of consciousness, functional connectivity between the PCC and both the GPe and CL was further analyzed. Results indicated that, compared to patients in the vegetative state and minimally conscious state groups, the coma group showed significantly reduced functional connectivity between the PCC and the GPe. Conversely, functional connectivity between the PCC and the bilateral central lateral thalamic (CL_L and CL_R) nuclei was significantly enhanced in the coma group. All above intergroup differences presented large effect sizes [*r* = 0.63–0.79, 95% CI = (0.32, 0.91), all *P* < 0.01]. For detailed findings (see Figures 3 and 4 and Tables 6 and 7).

**Table 6:** Brain regions showing altered FC with the PCC seed in coma and VS/MCS groups.

ROI	Brain region	AAL	MNI		
			X	Y	Z
PCC	Frontal_Sup_L	3	−22	68	02
	Frontal_Sup_R	4	18	42	48
	Frontal_Mid_R	8	26	12	50
	Temporal_Sup_R	82	52	−56	24
	Temporal_Mid_R	86	58	−14	−08
	Cingulum_Ant_L	31	−14	10	40
	Precentral_R	2	38	02	46

AAL, automated anatomical labeling atlas; MNI, Montreal Neurological Institute standard space.

**Table 7:** 95% CI of intergroup FC (PCC seed).

Functional connectivity pair	*r*	95% CI	*P*-value
PCC-AAL31	0.74	[0.47, 0.89]	< 0.001
PCC-AAL86	0.7	[0.41, 0.87]	< 0.001
PCC-AAL2	0.72	[0.44, 0.88]	< 0.001
PCC-AAL3,4	0.68	[0.39, 0.86]	< 0.001
PCC-AAL8	0.63	[0.32, 0.83]	< 0.01
PCC-AAL82	0.71	[0.42, 0.87]	< 0.001
PCC-GPe	0.69	[0.40, 0.86]	< 0.001
PCC-CL_L	−0.79	[−0.90, −0.53]	< 0.001
PCC-CL_R	−0.76	[−0.89, −0.49]	< 0.001

**Figure 5:**
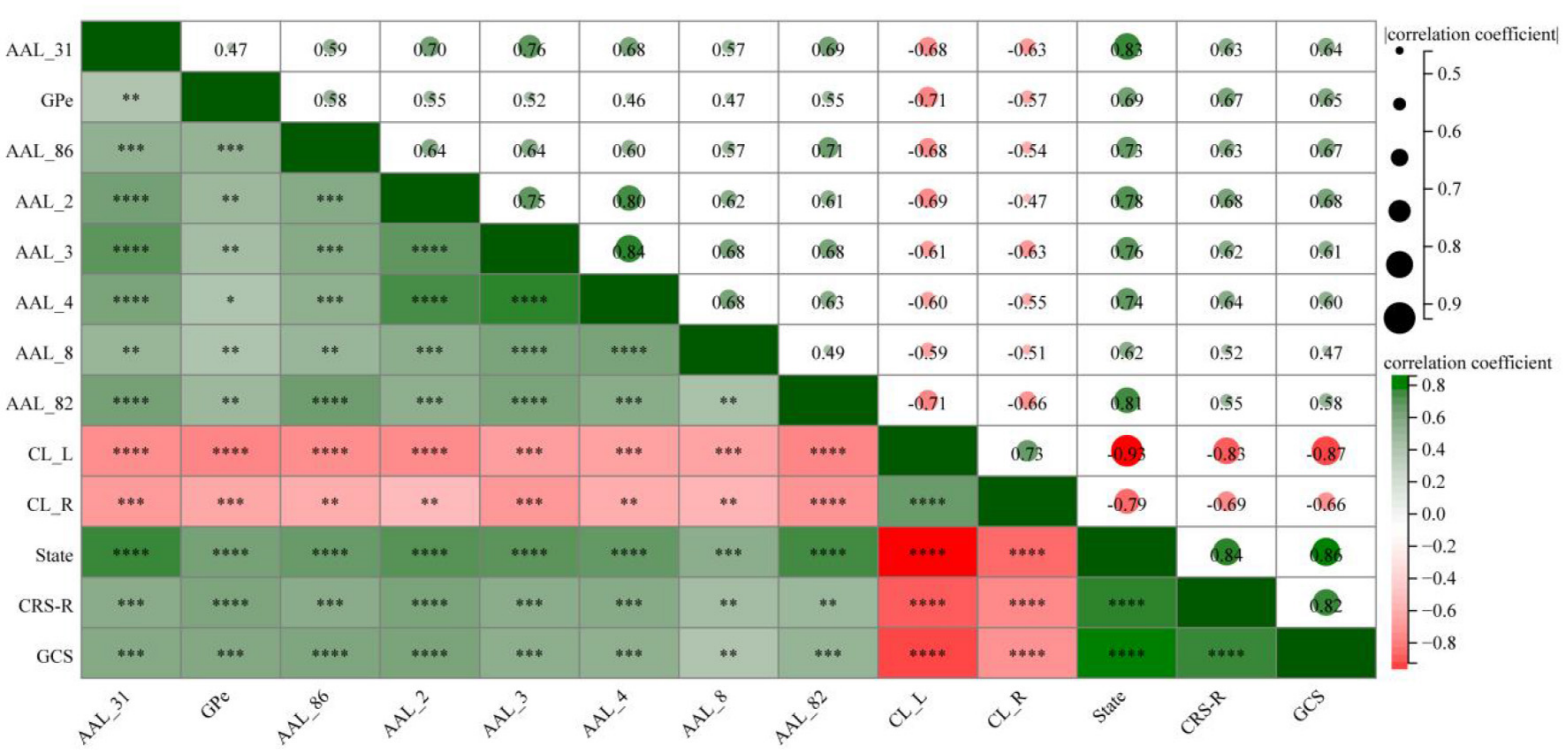
Correlation matrix between altered FC in brain regions and clinical assessments after FDR correction (using the PCC as a seed region). The horizontal and vertical axes are uniformly set as the brain regions with significantly altered functional connectivity based on PCC seed analysis. AAL_31: Cingulum Ant_L; Gpe: Globus Pallidus externa; AAL_86: Temporal_Mid_R; AAL2:Precentral_R; AAL3: Frontal_Sup_L; AAL4: Frontal_Sup_R; AAL8: Frontal_Mid_R; AAL82: Temporal_Sup_R; CL: Thalamus Central lateral. State: Arousal state. The correlation matrix, generated using Spearman's method, depicts pairwise associations among continuous variables, with point-biserial correlation used for relationships involving the dichotomous arousal state. Correlation strength is encoded through circle size (larger circles indicate stronger correlations) and color intensity based on a red-white-green (RWG) divergence scheme. Asterisks denote statistical significance levels after Benjamini–Hochberg false discovery rate (FDR) correction. ***P < 0.001; **P < 0.01; *P < 0.05; ****P < 0.0001. Non-significant correlations (*q* ≥ 0.05) are shown without asterisks.

Correlation analyses revealed significant positive associations between FC of the PCC and multiple clinical measures. PCC connectivity with the left anterior cingulate cortex strongly correlated with arousal (rs = 0.83, *P* < 0.0001), GCS (rs = 0.64, *P* < 0.001), and CRS-R (rs = 0.63, *P* < 0.001). PCC connectivity with the right middle temporal gyrus showed significant positive correlations with arousal (rs = 0.73, *P* < 0.0001), GCS (rs = 0.67, *P* < 0.001), and CRS-R (rs = 0.63, *P* < 0.0001). Strong correlations were also observed between PCC-right precentral gyrus connectivity and arousal (rs = 0.78, *P* < 0.0001), GCS (rs = 0.68, *P* < 0.0001), and CRS-R (rs = 0.68, *P* < 0.0001). PCC connectivity with the left and right superior frontal gyrus was significantly correlated with all three clinical measures (arousal: rs = 0.76 and 0.74, *P* < 0.0001; GCS: rs = 0.61, *P* < 0.001 and rs = 0.60, *P* < 0.001; CRS-R: rs = 0.62, *P* < 0.001 and rs = 0.64, *P* < 0.001). PCC-right middle frontal gyrus connectivity correlated with arousal (rs = 0.62, *P* < 0.001), GCS (rs = 0.47, *P* < 0.01), and CRS-R (rs = 0.52, *P* < 0.01). PCC-right superior temporal gyrus connectivity demonstrated positive correlations with arousal (rs = 0.81, *P* < 0.0001), GCS (rs = 0.58, *P* < 0.001), and CRS-R (rs = 0.55, *P* < 0.01). PCC–GPe connectivity exhibited strong positive correlations with arousal (rs = 0.69, *P* < 0.0001), GCS (rs = 0.65, *P* < 0.001), and CRS-R (rs = 0.67, *P* < 0.0001). PCC-CL_L connectivity was significantly negative correlated with arousal (rs = −0.93, *P* < 0.0001), GCS (rs = −0.87, *P* < 0.0001), and CRS-R (rs = −0.83, *P* < 0.0001). PCC-CL_R connectivity was significantly negative correlated with arousal (rs = −0.79, *P* < 0.0001), GCS (rs = −0.66, *P* < 0.0001), and CRS-R (rs = −0.69, *P* < 0.0001). The 95% confidence intervals of the above correlation coefficients ranged from [−0.97, −0.81] to [0.62, 0.93], with narrow interval widths indicating high reliability of the correlational results (see Figure 5 and Table 8). Statistical significance was defined as *P* < 0.05 after adjustment for multiple comparisons using the Benjamini–Hochberg false discovery rate (FDR) procedure. Correlation analyses revealed significant positive associations between the PCC and cortical regions. The PCC demonstrated significant positive correlations with the predefined regions of interest—the GPe within the “Mesocircuit” model. These correlations were particularly pronounced with respect to arousal levels. These findings further indicate a specific coupling between the default mode network and the “Mesocircuit” model, underscoring their important roles in the generation and recovery of consciousness. We further evaluated the prognostic value of functional connectivity strength in predicting arousal levels in patients.

**Table 8:** 95% CI for correlations (PCC-FC vs. clinical scores).

Functional connectivity pair	State rs	95% CI	GCS rs	95% CI	CRS-Rrs	95% CI	*P*-value
PCC-AAL31	0.83	[0.62, 0.93]	0.64	[0.33, 0.83]	0.63	[0.32, 0.82]	< 0.001
PCC-AAL86	0.73	[0.46, 0.89]	0.67	[0.37, 0.85]	0.63	[0.32, 0.82]	< 0.001
PCC-AAL2	0.78	[0.52, 0.91]	0.68	[0.38, 0.85]	0.68	[0.38, 0.85]	< 0.001
PCC-AAL3	0.76	[0.49, 0.90]	0.61	[0.29, 0.81]	0.62	[0.30, 0.82]	< 0.001
PCC-AAL4	0.74	[0.47, 0.89]	0.6	[0.28, 0.80]	0.64	[0.33, 0.83]	< 0.001
PCC-AAL8	0.62	[0.30, 0.82]	0.47	[0.13, 0.71]	0.52	[0.19, 0.75]	< 0.01
PCC-AAL82	0.81	[0.59, 0.92]	0.58	[0.26, 0.79]	0.55	[0.22, 0.77]	< 0.01
PCC-GPe	0.69	[0.40, 0.86]	0.65	[0.34, 0.83]	0.67	[0.37, 0.85]	< 0.001
PCC-CL_L	−0.93	[−0.97, −0.81]	−0.87	[−0.94, −0.69]	−0.83	[−0.92, −0.62]	< 0.001
PCC-CL_R	−0.79	[−0.90, −0.53]	−0.66	[−0.84, −0.36]	−0.69	[−0.86, −0.40]	< 0.001

**Figure 6:**
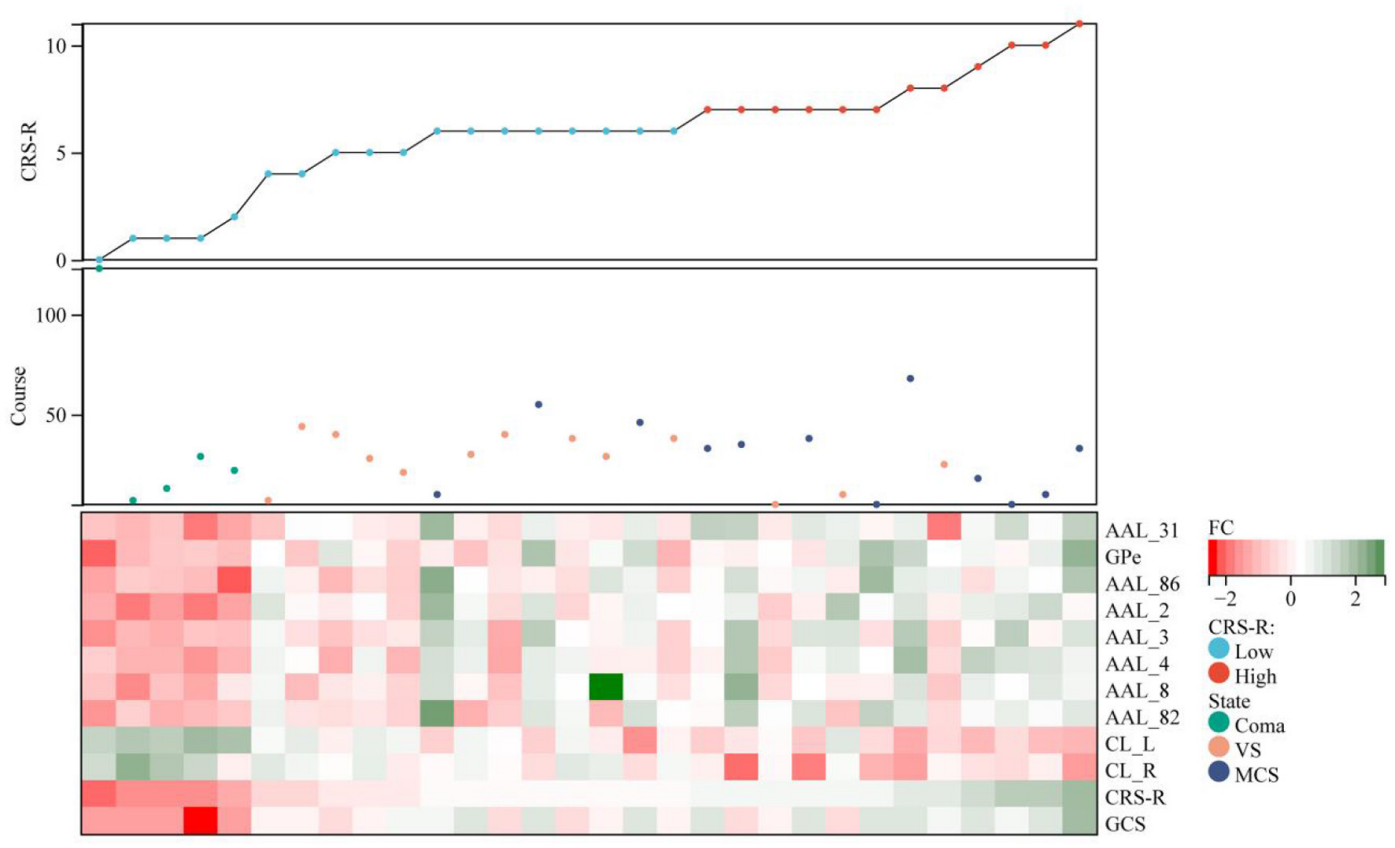
Heatmap of functional connectivity between the PCC and differential brain regions and its correlation with prognostic evaluation. AAL_31: Cingulum Ant_L; Gpe: Globus Pallidus externa; AAL_86: Temporal_Mid_R; AAL2:Precentral_R; AAL3: Frontal_Sup_L; AAL4: Frontal_Sup_R; AAL8: Frontal_Mid_R; AAL82: Temporal_Sup_R; CL: Thalamus Central lateral. The color gradient represents Fisher's Z-transformed correlation coefficients, with green tones indicating positive connectivity and red tones indicating negative connectivity. Course: Symptom onset to scan interval.

We examined the relationships between CRS-R scores and both the time from symptom onset to scanning and changes in functional connectivity strength using the PCC as a seed region. Details are presented in Figure 6. As shown in the top panel, higher CRS-R scores (from left to right on the x-axis) were associated with a greater proportion of patients in the MCS. Correspondingly, the time from symptom onset to scanning was significantly shorter in these patients, as illustrated in the middle panel. The strength of functional connectivity between the PCC and various differential brain regions exhibited an overall increasing trend with higher CRS-R scores, as demonstrated in the bottom panel. Notably, more pronounced changes were observed in AAL_86, AAL_2, AAL_3, and AAL_4, while more modest alterations were detected in GPe, AAL_8, AAL_82, and AAL_31. As expected, FC between the PCC and the mPFC (AAL_2, AAL_3, AAL_4, AAL_8) showed a positive correlation with CRS-R scores, suggesting its potential as a prognostic indicator. Notably, functional connectivity between the PCC and the bilateral central lateral thalamic nuclei (CL_L and CL_R) exhibited a negative correlation with improvements in consciousness levels.

#### 3.2.4 FC analysis using the mPFC as a seed region

**Figure 7:**
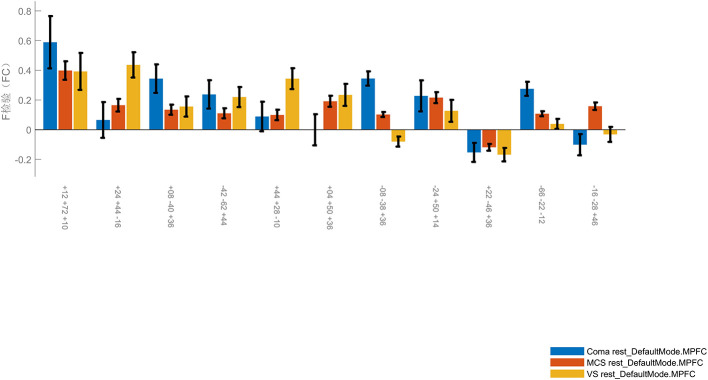
mPFC-seeded FC differences a coma/VS/MCS patient groups in significantly altered brain regions. (+12 +72 +10): Frontal sup R; (+24 +44 −16): Frontal Mid Orb R; (+8 −40 +36): Cingulum R; (−42 −62 +44): Angular L; (+44 +28 −10): Frontal Mid R; (+4 +50 +36): Frontal sup Medical R; (−08 −38 +36): Cingulum L; (−24 +50 +24): Frontal sup L; (+22 −46 +36): Cingulum R; (−66 −22 −12): Supramarginal L; (−16 −28 +46): Cingulum L. More pronounced *F*-values indicate greater differences in functional network organization across clinical groups (e.g., coma, VS, MCS).

**Figure 8:**
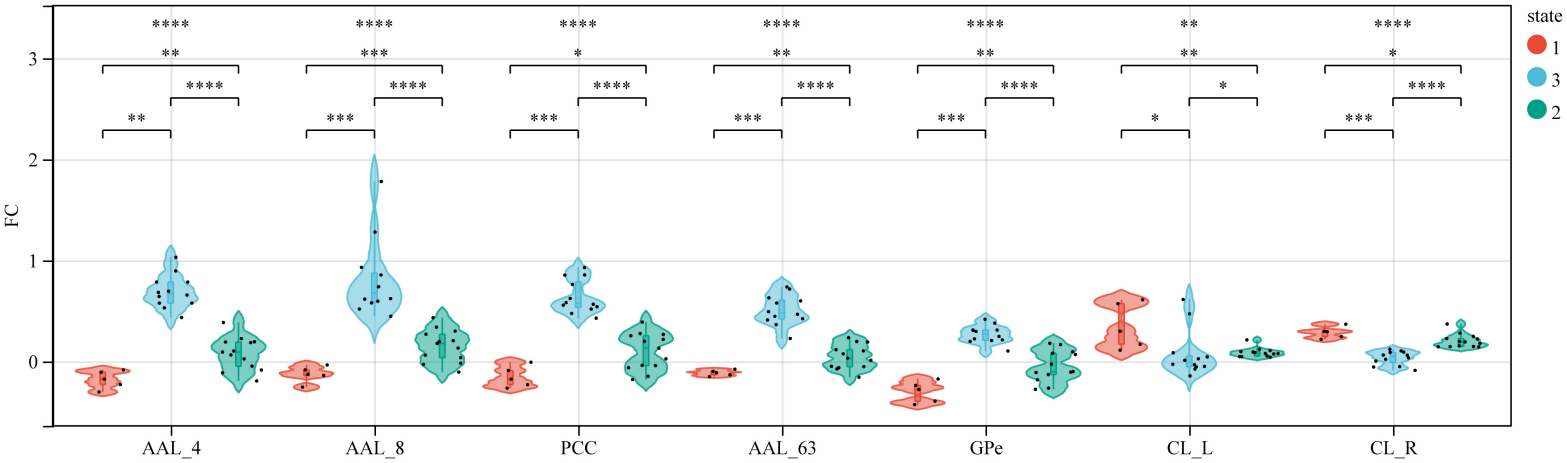
FC between the mPFC and specific brain regions across coma, VS, and MCS groups. Violin plots depict the distribution of FC between mPFC and seven specific brain regions across groups. AAL_4: Frontal_Sup_R; AAL_8: Frontal_Mid_R; AAL_63: SupraMarginal_L; GPe: Globus Pallidus externa; PCC: Posterior Cingulate Cortex; CL_L: left Thalamus Central lateral; CL_R: right Thalamus Central lateral. Differences in FC among the three groups were analyzed using non-parametric tests. ****P < 0.0001; ***P < 0.001; **P < 0.01; *P < 0.05. State: (1) Coma; (2) VS; (3) MCS.

Compared with the VS and MCS groups, patients in the coma group exhibited significantly reduced functional connectivity between the mPFC and several brain regions, including the right superior frontal gyrus (AAL_4: Frontal_Sup_R), right middle frontal gyrus (AAL_8: Frontal_Mid_R), left supramarginal gyrus (AAL_63: SupraMarginal_L), and the posterior cingulate cortex. These results survived Gaussian random field correction at a voxel-level threshold of *P* < 0.001 and a cluster-level threshold of *P* < 0.05. Further analysis of functional connectivity strength between the MPFC and the GPe, as well as bilateral CL (CL_L: left Thalamus Central lateral; CL_R: right Thalamus Central lateral), revealed that patients in the coma group showed significantly reduced MPFC-GPe connectivity compared to both the vegetative state and minimally conscious state groups. In contrast, enhanced functional connectivity was observed between the MPFC and bilateral CL in the coma group relative to the other two groups. These intergroup differences yielded medium to large effect sizes [*r* = −0.82 to 0.77, 95% CI = (−0.92, 0.90), all *P* < 0.05]. Details are provided in Figures 7 and 8 and Tables 9 and 10.

**Table 9:** Brain regions showing altered FC with the mPFC seed in coma and VS/MCS groups.

ROI	Brain region	AAL	MNI		
			X	Y	Z
mPFC	Frontal_Sup_R	4	12	72	10
	Frontal_Mid_R	8	44	28	−10
	Cingulum_L	35	−8	−38	36
	Cingulum_R	36	22	−46	36
	SupraMarginal_L	63	−66	−22	−12

AAL, automated anatomical labeling atlas; MNI, Montreal Neurological Institute standard space.

**Table 10:** 95% CI of intergroup FC (mPFC seed).

Functional connectivity pair	*r*	95% CI	*P*-value
mPFC-AAL4	0.77	[0.51, 0.90]	< 0.001
mPFC-AAL8	0.71	[0.42, 0.87]	< 0.001
mPFC-AAL63	0.76	[0.49, 0.89]	< 0.001
mPFC-PCC	0.79	[0.53, 0.91]	< 0.001
mPFC-GPe	0.77	[0.51, 0.90]	< 0.001
mPFC-CL_L	−0.41	[−0.68, −0.07]	< 0.05
mPFC-CL_R	−0.82	[−0.92, −0.58]	< 0.001

Correlation analyses revealed significant positive associations between functional connectivity of the mPFC and clinical measures. mPFC-right superior frontal gyrus connectivity strongly correlated with arousal (rs = 0.89, *P* < 0.0001), GCS (rs = 0.68, *P* < 0.0001), and CRS-R scores (rs = 0.69, *P* < 0.0001). mPFC-right middle frontal gyrus connectivity showed positive correlations with arousal (rs = 0.80, *P* < 0.0001), GCS (rs = 0.53, *P* < 0.01), and CRS-R (rs = 0.54, *P* < 0.01). Significant positive correlations were also observed between mPFC-PCC connectivity and arousal (rs = 0.87, *P* < 0.0001), GCS (rs = 0.71, *P* < 0.001), and CRS-R scores (rs = 0.64, *P* < 0.0001). mPFC-left supramarginal gyrus connectivity demonstrated strong positive correlations with arousal (rs = 0.87, *P* < 0.0001), GCS (rs = 0.68, *P* < 0.0001), and CRS-R (rs = 0.71, *P* < 0.0001). mPFC-GPe connectivity was also significantly positively correlated with arousal (rs = 0.87, *P* < 0.0001), GCS (rs = 0.68, *P* < 0.0001), and CRS-R scores (rs = 0.69, *P* < 0.0001).

**Figure 9:**
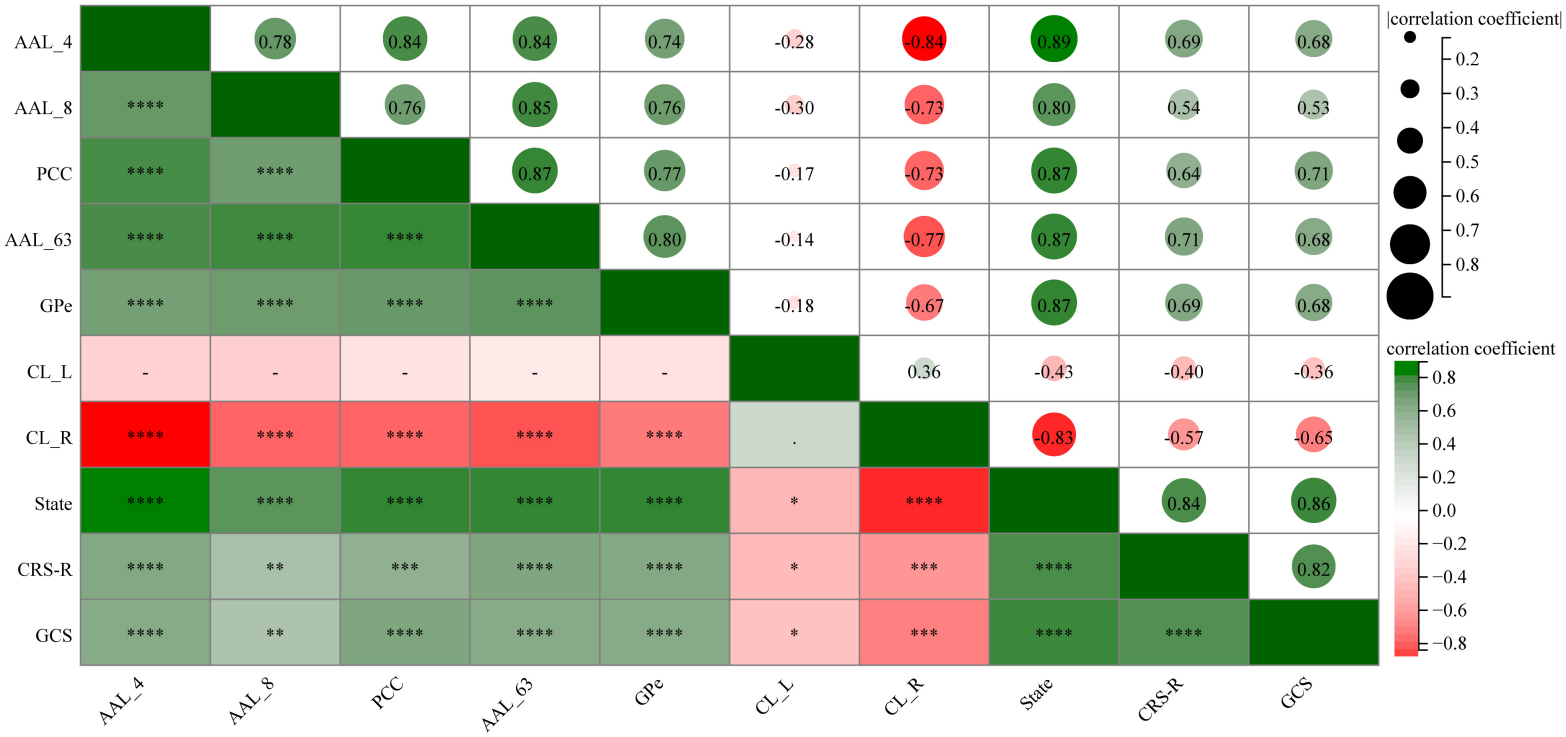
Correlation matrix between altered FC in brain regions and clinical assessments after FDR correction (using the mPFC as a seed region). The horizontal and vertical axes are uniformly set as the brain regions with significantly altered functional connectivity based on mPFC seed analysis. AAL_4: Frontal_Sup_R; AAL_8: Frontal_Mid_R; AAL_63: SupraMarginal_L; GPe: Globus Pallidus externa; PCC: Posterior Cingulate Cortex; CL_L: left Thalamus Central lateral; CL_R: right Thalamus Central lateral. State: Arousal state. The correlation matrix, generated using Spearman's method, depicts pairwise associations among continuous variables, with point-biserial correlation used for relationships involving the dichotomous arousal state. Correlation strength is encoded through circle size (larger circles indicate stronger correlations) and color intensity based on a red-white-green (RWG) divergence scheme. Asterisks denote statistical significance levels after Benjamini–Hochberg false discovery rate (FDR) correction. ***P < 0.001; **P < 0.01; *P < 0.05; ****P < 0.0001. Non-significant correlations (*q* ≥ 0.05) are shown without asterisks.

In contrast, negative correlations were identified between mPFC-left central lateral thalamic nucleus connectivity and arousal (rs = −0.43, *P* < 0.05), GCS (rs = −0.36, *P* < 0.05), and CRS-R scores (rs = −0.40, *P* < 0.05). Moreover, mPFC-right central lateral thalamic nucleus connectivity was significantly negatively correlated with arousal (rs = −0.83, *P* < 0.0001), GCS (rs = −0.65, *P* < 0.001), and CRS-R scores (rs = −0.57, *P* < 0.001). The 95% confidence intervals for above correlation coefficients were calculated as [−0.93, −0.09] to [0.72, 0.96], confirming the stability of these correlational relationships. Visualizations of these correlations are provided in Figure 9 and Table 11.

**Table 11:** 95% CI for correlations (mPFC vs. clinical scores).

Functional connectivity pair	State rs	95% CI	GCS rs	95% CI	CRS-R rs	95% CI	*P*-value
mPFC-AAL4	0.89	[0.72, 0.96]	0.68	[0.38, 0.85]	0.69	[0.40, 0.86]	< 0.001
mPFC-AAL8	0.8	[0.56, 0.92]	0.53	[0.19, 0.76]	0.54	[0.20, 0.77]	< 0.01
mPFC-PCC	0.87	[0.68, 0.95]	0.71	[0.42, 0.87]	0.64	[0.33, 0.83]	< 0.001
mPFC-AAL63	0.87	[0.68, 0.95]	0.68	[0.38, 0.85]	0.71	[0.42, 0.87]	< 0.001
mPFC-GPe	0.87	[0.68, 0.95]	0.68	[0.38, 0.85]	0.69	[0.40, 0.86]	< 0.001
mPFC-CL_L	−0.43	[−0.69, −0.09]	−0.36	[−0.64, −0.01]	−0.4	[−0.67, −0.06]	< 0.05
mPFC-CL_R	−0.83	[−0.93, −0.59]	−0.65	[−0.83, −0.34]	−0.57	[−0.78, −0.24]	< 0.001

**Figure 10:**
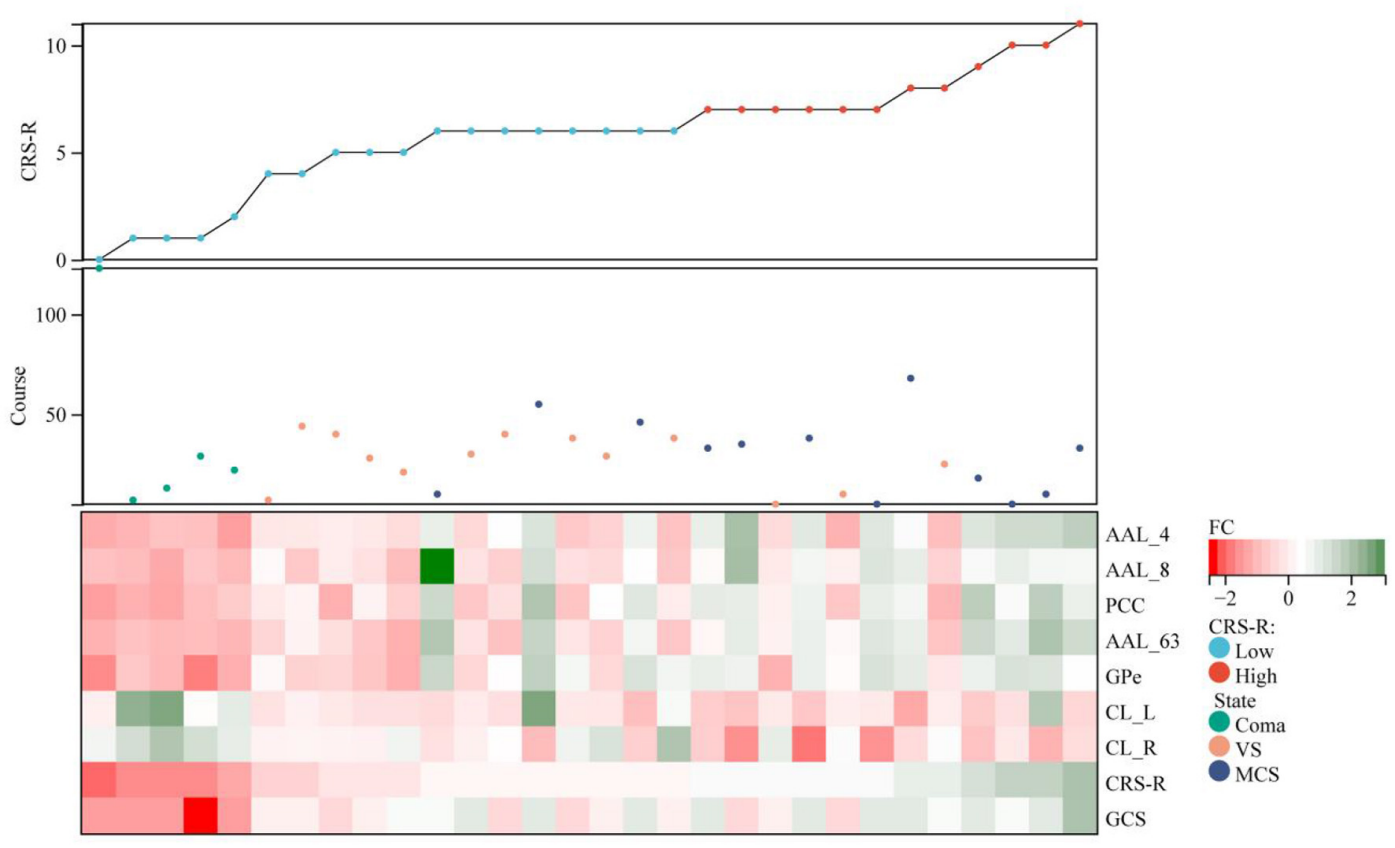
Heatmap of functional connectivity between the mPFC and differential brain regions and its correlation with prognostic evaluation. AAL_4: Frontal_Sup_R; AAL_8: Frontal_Mid_R; AAL_63: SupraMarginal_L; GPe: Globus Pallidus externa; PCC: Posterior Cingulate Cortex; CL_L: left Thalamus Central lateral; CL_R: right Thalamus Central lateral. The color gradient represents Fisher's Z-transformed correlation coefficients, with green tones indicating positive connectivity and red tones indicating negative connectivity. Course: Symptom onset to scan interval.

Analysis of heatmaps and prognostic correlations revealed that improvements in CRS-R scores were positively correlated with enhanced functional connectivity between the mPFC and regions including AAL_4, AAL_8, AAL_63, the PCC, and the GPe. Notably, the increases in connectivity between MPFC and AAL_4, PCC, and AAL_63 core nodes of the DMN were particularly pronounced. Conversely, mPFC connectivity with both the left and right central lateral thalamic nuclei (CL_L and CL_R), key components of the Mesocircuit model, exhibited a negative correlation with CRS-R scores, indicating a reduction in connectivity alongside clinical improvement (see Figure 10). These opposing trends suggest a coupled modulation between DMN integration and “Mesocircuit” Model throughout recovery of consciousness.

#### 3.2.5 FC analysis using the GPe as a seed region

**Figure 11:**
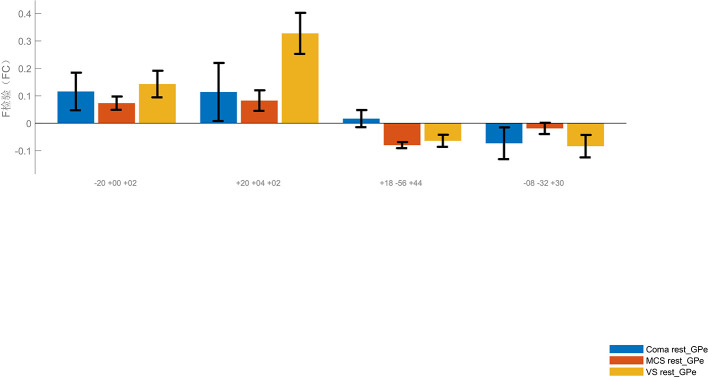
GPe-seeded FC differences a coma/VS/MCS patient groups in significantly altered brain regions. (−20 +0 +2): Palidum L; (+20 +4 +2): Palidum R; (+18 −56 +44): Precuneus R; (−08 −32 +30): Cingulum post L. More pronounced *F*-values indicate greater differences in functional network organization across clinical groups (e.g., coma, VS, MCS).

**Figure 12:**
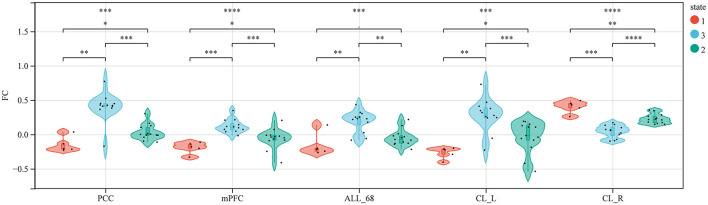
FC between the GPe and specific brain regions across coma, VS, and MCS groups. Violin plots depict the distribution of FC between GPe and five specific brain regions across groups. PCC: Posterior Cingulate Cortex; mPFC: medial prefrontal cortex; AAL_68: Precuneus R; CL_L: left Thalamus Central lateral; CL_R: right Thalamus Central lateral. Differences in FC among the three groups were analyzed using non-parametric tests. ****P < 0.0001; ***P < 0.001; **P < 0.01; *P < 0.05. State: (1) Coma; (2) VS; (3) MCS.

Comparative analysis using bilateral GPe as the seed region across the three patient groups revealed that, compared to patients in the vegetative state and minimally conscious state groups, those in the coma group exhibited significantly reduced functional connectivity between the GPe and several key regions of the DMN including the PCC, right precuneus (AAL_68 Precuneus_R), and mPFC—as well as the left central lateral thalamic nucleus, a component of the “Mesocircuit” Model. Conversely, the coma group showed enhanced functional connectivity between the GPe and the right central lateral thalamic nucleus, which also belongs to the “Mesocircuit” Model (Gaussian random field corrected, voxel-level *P* < 0.001, cluster-level *P* < 0.05). All intergroup comparisons presented large effect sizes [*r* = −0.86 to 0.76, 95% CI = (−0.94, 0.89), all *P* < 0.001]. Details are provided in Figures 11 and 12 and Tables 12 and 13. These differential connectivity patterns suggest a potential decoupling between DMN integration and “Mesocircuit” Model in patients with coma, which may reflect impaired consciousness and poor prognostic outcomes. Using the GPe as a seed region, correlation analyses were conducted to examine functional connectivity with brain regions showing significant group differences, as well as predefined regions of interest within the DMN and the “Mesocircuit” Model.

**Table 12:** Brain regions showing altered FC with the GPe seed in coma and VS/MCS groups.

ROI	Brain region	AAL	MNI		
			X	Y	Z
GPe	Precuneus_R	68	18	−56	44
	Cingulum_L	35	−8	−32	30

AAL, automated anatomical labeling atlas; MNI, Montreal Neurological Institute standard space.

**Table 13:** 95% CI of intergroup FC (GPe seed).

Functional connectivity pair	*r*	95% CI	*P*-value
GPe-PCC	0.76	[0.49, 0.89]	< 0.001
GPe-mPFC	0.72	[0.44, 0.88]	< 0.001
GPe-AAL68	0.7	[0.41, 0.87]	< 0.001
GPe-CL_L	0.7	[0.41, 0.87]	< 0.001
GPe-CL_R	−0.86	[−0.94, −0.64]	< 0.001

**Figure 13:**
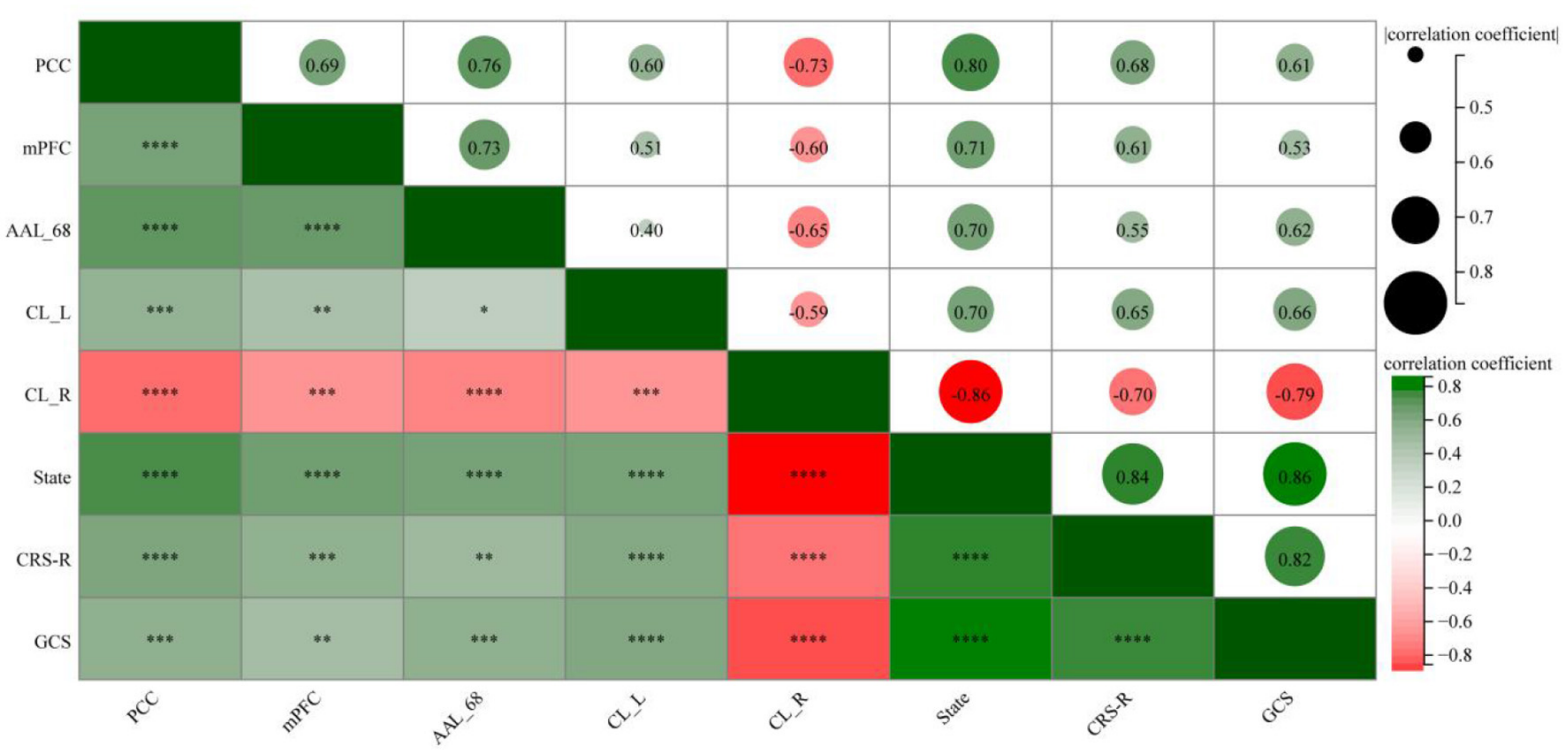
Correlation matrix between altered FC in brain regions and clinical assessments after FDR correction (using the GPe as a seed region). The horizontal and vertical axes are uniformly set as the brain regions with significantly altered functional connectivity based on GPe seed analysis. PCC: Posterior Cingulate Cortex; mPFC: medial prefrontal cortex; AAL_68: Precuneus R; CL_L: left Thalamus Central lateral; CL_R: right Thalamus Central lateral. State: Arousal state. The correlation matrix, generated using Spearman's method, depicts pairwise associations among continuous variables, with point-biserial correlation used for relationships involving the dichotomous arousal state. Correlation strength is encoded through circle size (larger circles indicate stronger correlations) and color intensity based on a red-white-green (RWG) divergence scheme. Asterisks denote statistical significance levels after Benjamini–Hochberg false discovery rate (FDR) correction. ***P < 0.001; **P < 0.01; *P < 0.05; ****P < 0.0001. Non-significant correlations (*q* ≥ 0.05) are shown without asterisks.

Significant correlations were observed between functional connectivity of the GPe and multiple clinical measures, with distinct patterns aligning with nodes of the DMN and the “Mesocircuit” Model. Strong positive correlations were identified between GPe-PCC connectivity and arousal (rs = 0.80, *P* < 0.0001), GCS (rs = 0.61, *P* < 0.0001), and CRS-R scores (rs = 0.68, *P* < 0.001). Similarly, GPe–mPFC connectivity correlated positively with arousal (rs = 0.71, *P* < 0.0001), GCS (rs = 0.53, *P* < 0.001), and CRS-R scores (rs = 0.61, *P* < 0.01). GPe-right precuneus connectivity also showed positive associations with arousal (rs = 0.70, *P* < 0.0001), GCS (rs = 0.62, *P* < 0.01), and CRS-R scores (rs = 0.55, *P* < 0.001). Notably, GPe–CL_L connectivity was positively correlated with arousal (rs = 0.70, *P* < 0.0001), GCS (rs = 0.66, *P* < 0.0001), and CRS-R scores (rs = 0.65, *P* < 0.0001). In contrast, GPe-CL_R connectivity demonstrated significant negative correlations with arousal (rs = −0.86, *P* < 0.0001), GCS (rs = −0.79, *P* < 0.0001), and CRS-R scores (rs = −0.70, *P* < 0.0001). The 95% confidence intervals of these correlation coefficients ranged from [−0.94, −0.53] to [0.56, 0.92], indicating reliable correlational results. For detailed visualizations (see Figure 13 and Table 14). These opposing correlation profiles between GPe–DMN and GPe-“Mesocircuit” Model suggest a coupled relationship between DMN integration and “Mesocircuit” Model underlying behavioral recovery in DoC.

**Table 14:** 95% CI for correlations (GPe vs. clinical scores).

Functional connectivity pair	State rs	95% CI	GCS rs	95% CI	CRS-R rs	95% CI	*P*-value
GPe-PCC	0.8	[0.56, 0.92]	0.61	[0.29, 0.81]	0.68	[0.38, 0.85]	< 0.001
GPe-mPFC	0.71	[0.42, 0.87]	0.53	[0.19, 0.76]	0.61	[0.29, 0.81]	< 0.01
GPe-AAL68	0.7	[0.41, 0.87]	0.62	[0.30, 0.82]	0.55	[0.22, 0.77]	< 0.001
GPe-CL_L	0.7	[0.41, 0.87]	0.66	[0.36, 0.84]	0.65	[0.34, 0.83]	< 0.001
GPe-CL_R	−0.86	[−0.94, −0.64]	−0.79	[−0.90, −0.53]	−0.7	[−0.87, −0.41]	< 0.001

**Figure 14:**
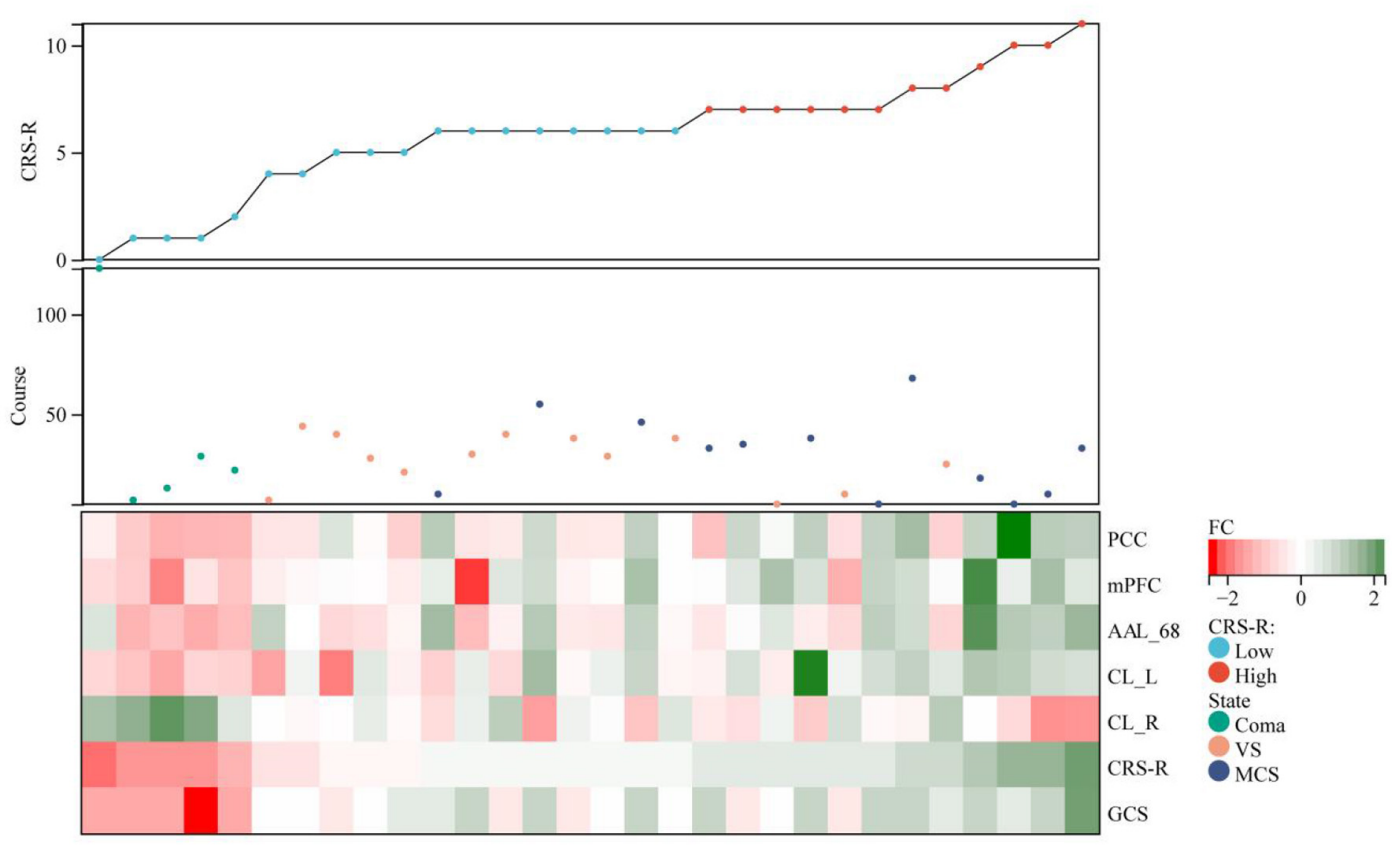
Heatmap of functional connectivity between the GPe and differential brain regions and its correlation with prognostic evaluation. PCC: Posterior Cingulate Cortex; mPFC: medial prefrontal cortex; AAL_68: Precuneus_R; CL_L: left Thalamus Central lateral; CL_R: right Thalamus Central lateral. The color gradient represents Fisher's Z-transformed correlation coefficients, with green tones indicating positive connectivity and red tones indicating negative connectivity. Course: Symptom onset to scan interval.

Analysis of heatmaps and prognostic correlations revealed that improvements in CRS-R scores were associated with enhanced functional connectivity between the GPe and several key regions of the default mode network (DMN), including AAL_68, the mPFC, and the PCC (see Figure 14). Among these, the strengthening of GPe-PCC connectivity was particularly pronounced. A moderate increasing trend was also observed between the GPe and the CL_L. By contrast, functional connectivity between the GPe and the CL_R exhibited a marked decrease as CRS-R scores improved. These distinct and opposing connectivity trends suggest a coupled involvement of DMN integration and “Mesocircuit” Model in the recovery of consciousness, with PCC connectivity serving as a prominent correlate of clinical improvement.

## 4 Discussion

### 4.1 Summary of key findings

This article takes DoC as the core content and conducts a relatively comprehensive review of the variation characteristics of functional connection strength between two particularly crucial neural networks, namely the DMN and the “Mesocircuit” Model, for patients with varying degrees of consciousness disorders. This research aims to clarify the connection between the operational capacity of neural networks and the degree of consciousness. In this study, rs-fMRI was employed as a precise neuroimaging tool to evaluate differences in functional connectivity among patients with varying DoC. Seed-based analysis was conducted using core regions of the DMN and the “Mesocircuit” Model—specifically, the PCC, MPFC, and GPe. FC patterns were systematically compared across three patient groups: coma, VS, and MCS. Analysis of the results revealed marked alterations in neural functional connectivity across all three patient groups. Significant disruptions were observed both within and between the major intrinsic networks, the integrity of functional connections demonstrated a progressive decline that correlated with the degree of consciousness impairment. The results obtained from these studies provide direct neuroimaging evidence for the theoretical claim that the default mode network and the Mesocircuit model exhibit a state of cooperative integration during the process of consciousness initiation, maintenance, and control. We also pointed out that functional connectivity measurements mainly focus on sites such as PCC, mPFC and GPe, which can serve as potential neurobiological markers for clinical diagnosis, classification of patients with consciousness disorders, and assessment of their prognosis. In this way, it provides some foundation for personalized medicine in this field and can also play a certain guiding role.

Consistent with our initial hypothesis, we obtained four key sets of findings: (1) Abnormal functional connectivity within core nodes of the DMN relative to the level of consciousness: compared with patients in the vegetative state and minimally conscious state groups, those in the coma group exhibited significantly reduced FC between core DMN nodes—specifically the PCC and mPFC—and various cortical and subcortical regions. In patients with coma, the functional connectivity between the PCC and the left anterior cingulate cortex, right middle temporal gyrus, and right precentral gyrus was significantly reduced. A similar decreasing trend was observed between the mPFC and both the right superior frontal gyrus and left supramarginal gyrus. The strongest connectivity across these regions was consistently identified in patients with MCS. (2) Decoupling between the DMN and the “Mesocircuit” Model: as key components of the central thalamocircuit model, the GPe and the bilateral central lateral thalamic nuclei (CL_L and CL_R) exhibited distinct patterns of functional connectivity with core regions of the DMN in comatose patients. Specifically, significantly reduced functional connectivity was observed between the GPe and DMN hubs—including the PCC, mPFC, and right precuneus. In contrast, enhanced functional connectivity was found between the bilateral CL and these same DMN regions within the same patient group. (3) Abnormal local neural activity associated with the level of consciousness: Comatose patients exhibited significantly lower raw ALFF in the bilateral CL, GPe, PCC, and mPFC compared to both the VS group (*P* < 0.001) and the MCS group (*P* < 0.001). VS patients showed reduced raw ALFF values in these regions relative to the MCS group (*P* < 0.001). (4) FC of core nodes in the DMN and “Mesocircuit” Model correlates with clinical measures of consciousness and arousal: FC between PCC-mPFC, PCC-GPe, and mPFC-GPe showed significant positive correlations with CRS-R scores. In contrast, connectivity between the bilateral CL and both PCC and mPFC was negatively correlated with CRS-R scores. (5) All significant group differences and correlation analyses presented large effect sizes with narrow 95% confidence intervals, further verifying the reliability of these potential biomarkers. A positive correlation was observed between GPe-CL_L connectivity and CRS-R scores, whereas GPe-CL_R connectivity demonstrated a significant negative correlation with CRS-R scores. These findings highlight that the interaction between the DMN and the “Mesocircuit” model has been disrupted, rather than an independent defect within a single network. This is the fundamental cause of functional impairment in patients with DoC. These results demonstrate that the DMN—“Mesocircuit” model plays a very crucial role in promoting the formation of conscious perception. We also suggest that these models may be used as neuroimaging biomarkers to predict what the clinical outcomes of this group of patients will be.

### 4.2 Altered FC among core hubs within the DMN

The DMN serves as a functionally integrated system that plays a pivotal role in self-referential processing, memory retrieval, social cognition, and theory of mind (32). Moreover, the functional integrity of the DMN has long been postulated to be a core mechanism underlying the sustenance of consciousness (33). Bodien et al. (16) initially demonstrated that, compared to patients in a VS, those in coma exhibited an overall reduction in functional connectivity within the DMN, whereas patients in a MCS retained partial integrity of the DMN this finding was further supported by Lutkenhoff et al. (14). As a core hub of the DMN, the PCC and its structural and functional connectivity have been extensively studied in patients with disorders of consciousness (34, 35). It plays a critical integrative role and maintains robust functional connections with other key DMN regions, such as the mPFC and the precuneus (36). Lemaire et al. (37) suggested that the potential restoration of impaired functional connectivity between the mPFC and PCC, as observed in patients with DoC, may serve as a prognostic marker for clinical recovery a finding consistent with our results.

In this study, this paper took PCC and mPFC as the centers and conducted a seed-based FC analysis. This analysis provided verification for the relevant conclusions. The results obtained from the study show that there is a very prominent correlation between DMN functional integration and consciousness level in patients with DoC. Within the network, compared with the VS group and the MCS group, the coma group showed a very significant weakening of functional connections between the PCC and multiple key regions. These regions mentioned here include the left anterior cingulate cortex, the right superior temporal gyrus, the right middle temporal gyrus, and the mPFC (comprising bilateral superior frontal gyri and the right middle frontal gyrus) this difference is statistically significant (*P* < 0.001). The strength of functional connectivity between the PCC and these cortical regions showed significantly positive correlations with both CRS-R and GCS scores (rs = 0.64–0.87, *P* < 0.001). These findings provide some support for the following view: the completeness of functional integration among many nodes in the DMN, is directly related to the degree of consciousness disorder, that is, the degree of consciousness of DoC patients. It is easy to see that as the patient's consciousness damage becomes more and more serious, the collapse of this comprehensive functional ability will become more and more obvious. Our findings demonstrated that the strength of functional connectivity between the PCC and mPFC showed the strongest correlation with arousal state (rs = 0.87, *P* < 0.0001), suggesting its potential role as an early marker of recovery.

In terms of cross-network interactions, the functional connectivity between the PCC/mPFC and the GPe also exhibited consciousness-level-dependent differences (38), with a significantly reduced strength in the coma group compared to the other groups (*P* < 0.001). GPe regulates cortical function through the basal ganglio-thalamic-cortical circuit. Such a rule provides more support for the central hypothesis of this study, which holds that the basal ganglio-thalamic-cortical circuit and the default mode network, namely DMN, The connection between them is very crucial for consciousness (11). These findings suggest that positive functional integration within the DMN and its coupling with the central loop are essential for the maintenance of consciousness. This finding aligns with and corroborates the research results reported by Edlow et al. (39), Qin et al. (40), and their respective teams. The strength of functional connectivity between the PCC/mPFC and the bilateral CL exhibited a progressive increase with the severity of consciousness impairment, demonstrating a negative correlation with clinical scores. This discovery might indicate that during a coma, the hypothalamic-cortical circuit undergoes pathological overstimulation. Under such circumstances, the defective regulatory process is unable to properly regulate the cortical network, which is crucial for maintaining consciousness (41). CL projects to the frontal cortex and may disrupt DMN activity through an excitatory-inhibitory imbalance, thereby hindering the integration of conscious content. This interpretation aligns with the “Mesocircuit” model, which posits that reduced thalamocortical drive disrupts global network integration and leads to loss of consciousness (42). Research by the team led by Demertzi et al. (42) indicates that anti-correlated FC between the DMN and other brain networks may represent a specific physiological marker for the emergence and recovery of consciousness. This pattern is thought to reflect the brain's capacity to selectively integrate key functional networks while suppressing task-irrelevant neural activity during conscious states—a regulatory process likely mediated by neural inhibitory mechanisms (42, 43). Our results are highly consistent with this theoretical framework. Our results further revealed that the strength of functional connectivity between the PCC and GPe exhibited the strongest correlation with CRS-R scores (rs = 0.67, *P* < 0.0001), suggesting its potential as a predictive biomarker for consciousness recovery. Conversely, increased connectivity between the PCC and CL may indicate a poor prognosis.

### 4.3 Altered FC among core hubs within the “Mesocircuit” Model

The GPe is regarded by everyone as a particularly complex neural structure. It not only has extensive interconnections with other nuclei of the basal ganglia but also has a direct connection with the cerebral cortex (44–47). Through its inhibitory efferents, the GPe contributes to the fine-tuned balance of neural circuits (48, 49). As a non-specific thalamic nucleus, the CL plays a critical role in cortico-subcortical information transfer and the regulation of arousal (50, 51). It is actually a particularly crucial node in the central thalamic-cortical circuit. Functionally speaking, it can connect subcortical components such as the basal ganglia with cortical regions associated with consciousness, such as the prefrontal cortex, which is a cortical region related to consciousness (52–55). According to the “Mesocircuit” model hypothesis proposed by Schiff, the DoC caused by severe brain injury is essentially due to the interference of cortical and subcortical signals within this specific circuit or the occurrence of functional disorders (52). Employing seed-based FC analysis with the GPe, this study systematically revealed abnormal connectivity patterns among core nodes of the “Mesocircuit” model across varying degrees of DoC using rs-fMRI. The results obtained from these studies provide very precise data support for this hypothesis and also enable us to have a better understanding of the pathophysiological processes hidden behind DoC.

Within the network, the coma group exhibited significantly reduced FC between the GPe and the CL_L compared to both the VS and MCS groups. The strength of this FC showed strong positive correlations with CRS-R scores (rs = 0.65, *P* < 0.0001), GCS scores (rs = 0.66, *P* < 0.0001), and the level of arousal (rs = 0.70, *P* < 0.0001). These findings suggest that the functional integrity of the GPe–CL_L pathway may be essential for the efficient transmission of subcortical signals to consciousness-related cortical regions such as the frontal lobe. Proper functional coupling within this pathway appears to support the organized propagation of arousal-related signals. The observed reduction in FC within this pathway under coma may directly contribute to decreased excitability in the prefrontal cortex, ultimately manifesting as diminished arousal and loss of conscious content. As consciousness levels improved, a progressive enhancement of FC between the GPe and CL_L was observed, further supporting the proposed facilitatory role of the “Mesocircuit” model in the process of consciousness recovery.

In contrast, the coma group demonstrated a significantly stronger FC strength between the GPe and the CL_R compared to both the VS and MCS groups (*P* < 0.001). This enhanced FC exhibited strong negative correlations with CRS-R scores (rs = −0.70, *P* < 0.0001) and GCS scores (rs = −0.79, *P* < 0.0001). This reverse aberrant pattern is not coincidental: Research by Zheng et al. (49) revealed a hemispheric specialization in the regulatory influence of the GPe over the thalamus: the GPe-CL_L pathway appears to facilitate the transmission of cognitive and arousal-related signals, whereas the GPe-CL_R pathway is predominantly involved in inhibitory modulation. The observed excessive enhancement of FC between the GPe and CL_R in this study is speculated to reflect an underlying dysregulation of subcortical inhibitory control during coma. Employing a reverse verification approach with the “Mesocircuit” model node GPe as a seed region during network interaction analysis, the results further corroborate—from the perspective of “Mesocircuit” model nodes—that the aberrant coupling between the DMN and the “Mesocircuit” model is bidirectional. Specifically, not only is signal reception from the DMN to the “Mesocircuit” model compromised, but efferent signaling from the “Mesocircuit” model to the DMN is also significantly impaired.

## 5 Limitations and future directions

This research is plagued by many limiting factors. Although the sample size is relatively small, all observed differences showed large effect sizes and narrow 95% confidence intervals, which indicate that the findings are statistically robust. The identified FC pairs are therefore interpreted as preliminary potential biomarkers for DoC diagnosis and prognosis. When we take into account the consciousness disorder, that is, the specific subgroup within the DoC, this situation may reduce the statistical strength and also lower the applicability of the results in a broader context. Because a cross-sectional research design was adopted, it is impossible for us to draw a causal conclusion about the connection between changes in FC and the path of consciousness recovery. That is to say, it is impossible to determine whether the changes in FC are the cause of alterations in the path of consciousness recovery or whether the situation of the path of consciousness recovery will have an impact on the changes in functional connectivity. At present, it is not possible to draw such a causal conclusion. Although the analysis we conducted did not find statistical differences in etiology among subgroups, there might be some heterogeneity in patients whose etiologies were combined. Another limitation of the present study is the heterogeneity of enrolled patients. The cohort simultaneously contained patients with traumatic brain injury (TBI) and non-traumatic brain injury (NTBI), and significant intergroup differences were observed in the time from disease onset to MRI scanning (*P* = 0.005). Different brain injury mechanisms (traumatic vs. non-traumatic) may lead to distinct patterns of neural circuit damage, while varying disease courses could alter the degree of spontaneous neural activity and interregional functional coupling in a time-dependent manner. These two confounding factors may interfere with the consistency of intergroup comparisons of ALFF and functional connectivity results.

Ideally, injury type and disease duration should be incorporated as covariates in a sensitivity analysis to eliminate their confounding effects and further validate the robustness of our core findings. Although subgroup stratified analysis was not performed in the current study due to the limited sample size, all core between-group differences exhibited large effect sizes with narrow 95% confidence intervals, which partially supported the reliability of the observed associations between network coupling and consciousness levels. Future work will expand the sample capacity and conduct sensitivity analysis adjusted for injury type and disease course to exclude such interference factors and strengthen the persuasiveness of our conclusions.

The examination in this article mainly focuses on the aspect of linear correlations. However, these linear correlations may not fully reflect the complex nonlinear mechanisms that form the basis of network interactions.

In the subsequent research work to be carried out, it is advisable to prioritize the inclusion of a larger sample size. Here, it is recommended to adopt a longitudinal research design approach, with a tracking period of 6–12 months as the standard. This research method is extremely crucial for verifying the predictive value of FC, that is, the FC indicator. Future research should adopt a multimodal framework that integrates fMRI with electrophysiological measures, diffusion tensor imaging (DTI), and biochemical markers. Leveraging machine learning to synthesize these multimodal data could significantly enhance the accuracy of diagnosis and prognosis in DoC, while further refining the interpretability and utility of FC analyses.

## 6 Conclusion

In summary, the research of this paper shows that the functional completeness of the DMN, the interconnection within the “Mesocircuit” model, and the interaction among these networks are all closely related to the consciousness level of patients with DoC. This study found the measurement of functional connectivity in key structural combinations such as PCC-GPe, PCC-mPFC, GPe-CL_L, and PCC-CL. It can be regarded as a prospective neurobiological indicator for the diagnosis of consciousness disorders and prognosis assessment. Subsequent research is well-grounded to conduct a more comprehensive assessment. These functional connectivity strengths can be used as reliable biomarkers, which can enhance the diagnosis of consciousness disorders and also improve the evaluation of the prognosis of consciousness disorders.

## Data Availability

The raw data supporting the conclusions of this article will be made available by the authors, without undue reservation.
